# Supplementary figures and images for: Morphology-dependent entry kinetics and spread of influenza A virus
Source: EMBO J. 2025 Jun 9;44(14):3959–82. doi: 10.1038/s44318-025-00481-6 (PMC12264294; doi:10.1038/s44318-025-00481-6)

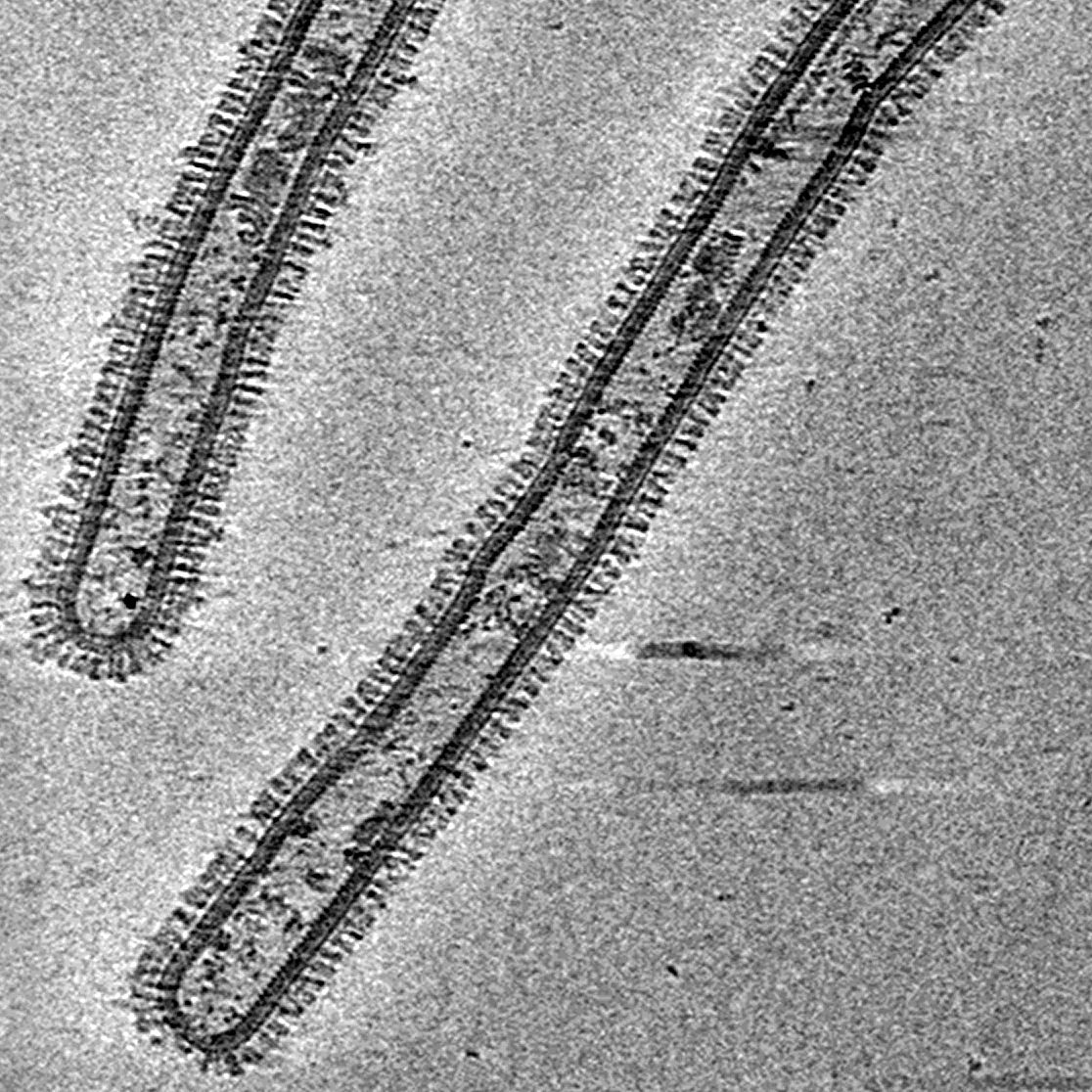

Supplement: Supplementary file 2 — Source data Fig. 1 [file 44318_2025_481_MOESM2_ESM.zip › Figure_01/1E/WSN-M1-Udorn.jpg]

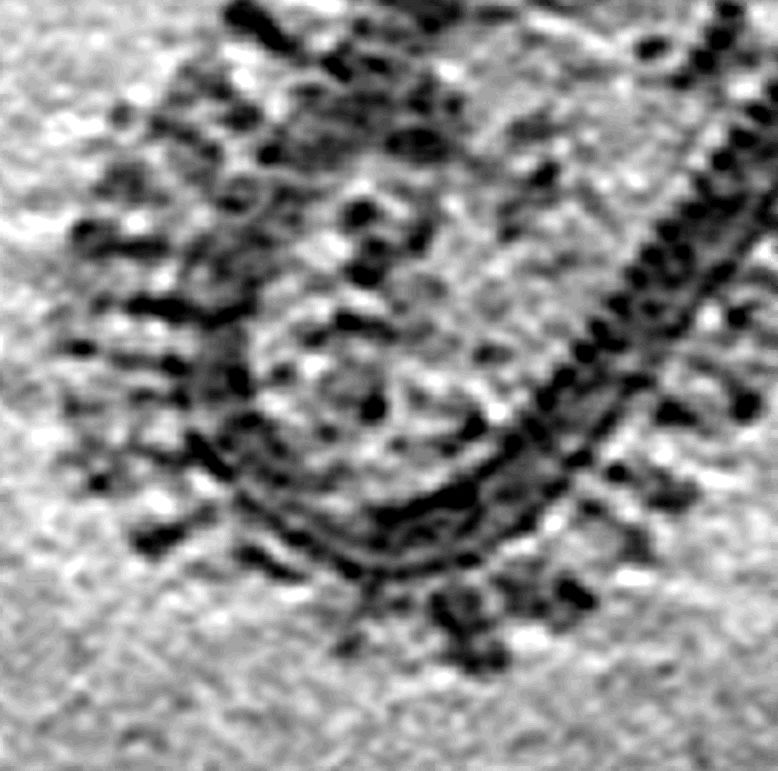

Supplement: Supplementary file 2 — Source data Fig. 1 [file 44318_2025_481_MOESM2_ESM.zip › Figure_01/1E/WSN-M1-Udorn-zoom_NA.jpg]

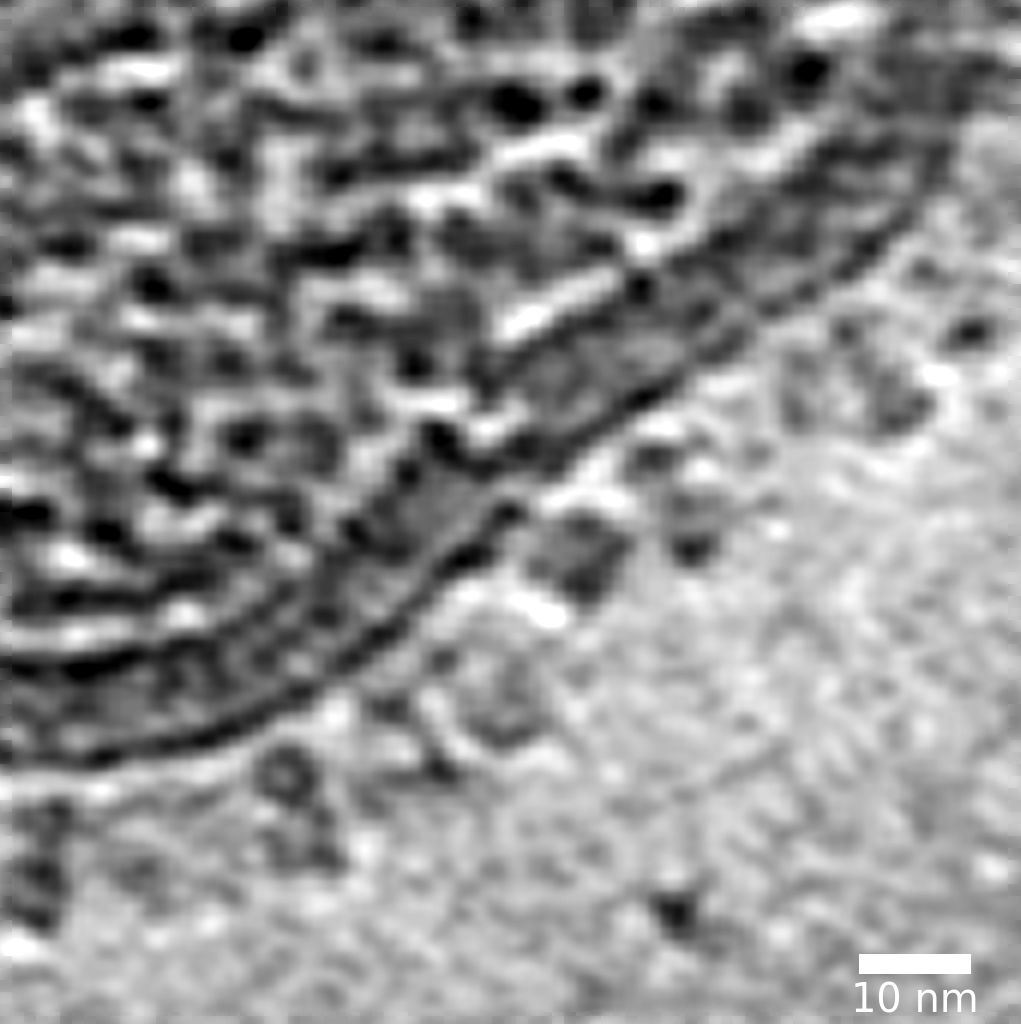

Supplement: Supplementary file 2 — Source data Fig. 1 [file 44318_2025_481_MOESM2_ESM.zip › Figure_01/1D/WSN-zoom-M1.jpg]

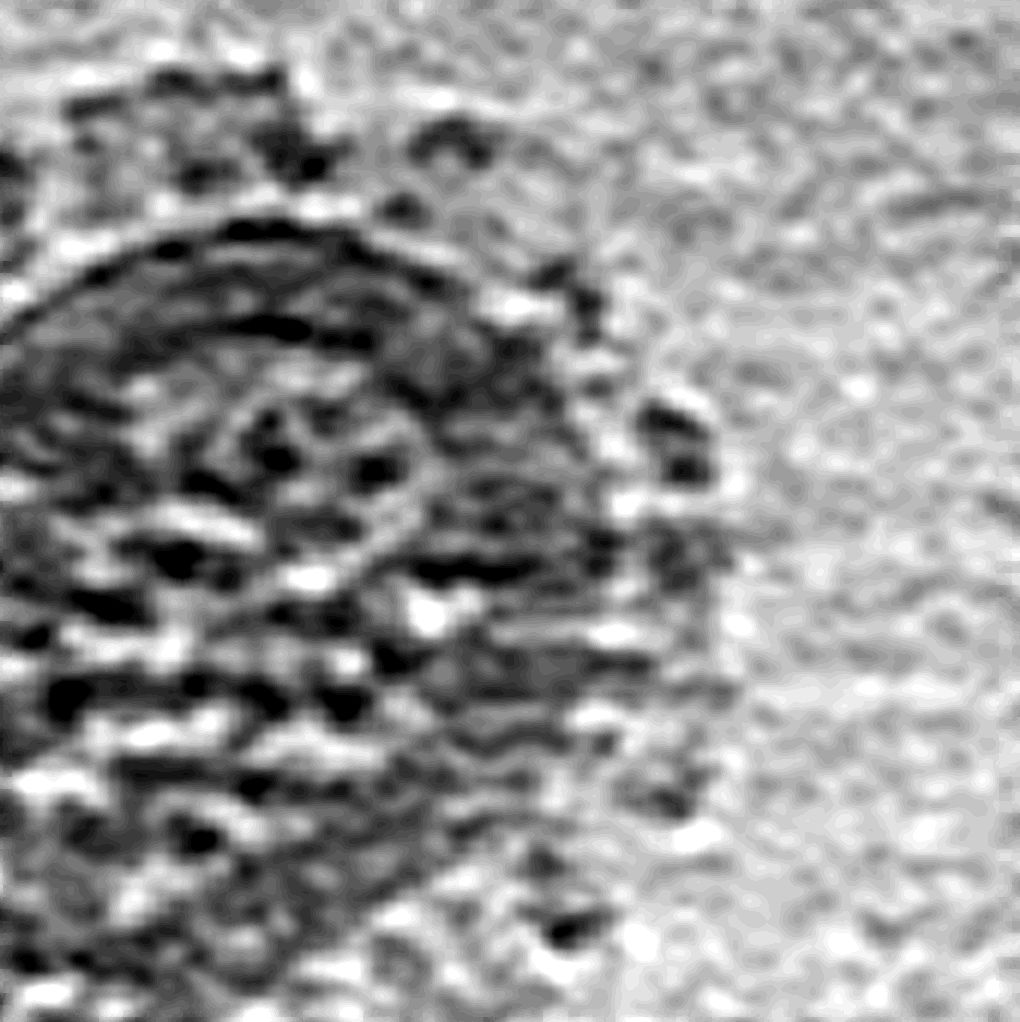

Supplement: Supplementary file 2 — Source data Fig. 1 [file 44318_2025_481_MOESM2_ESM.zip › Figure_01/1D/WSN-zoom_NA.jpg]

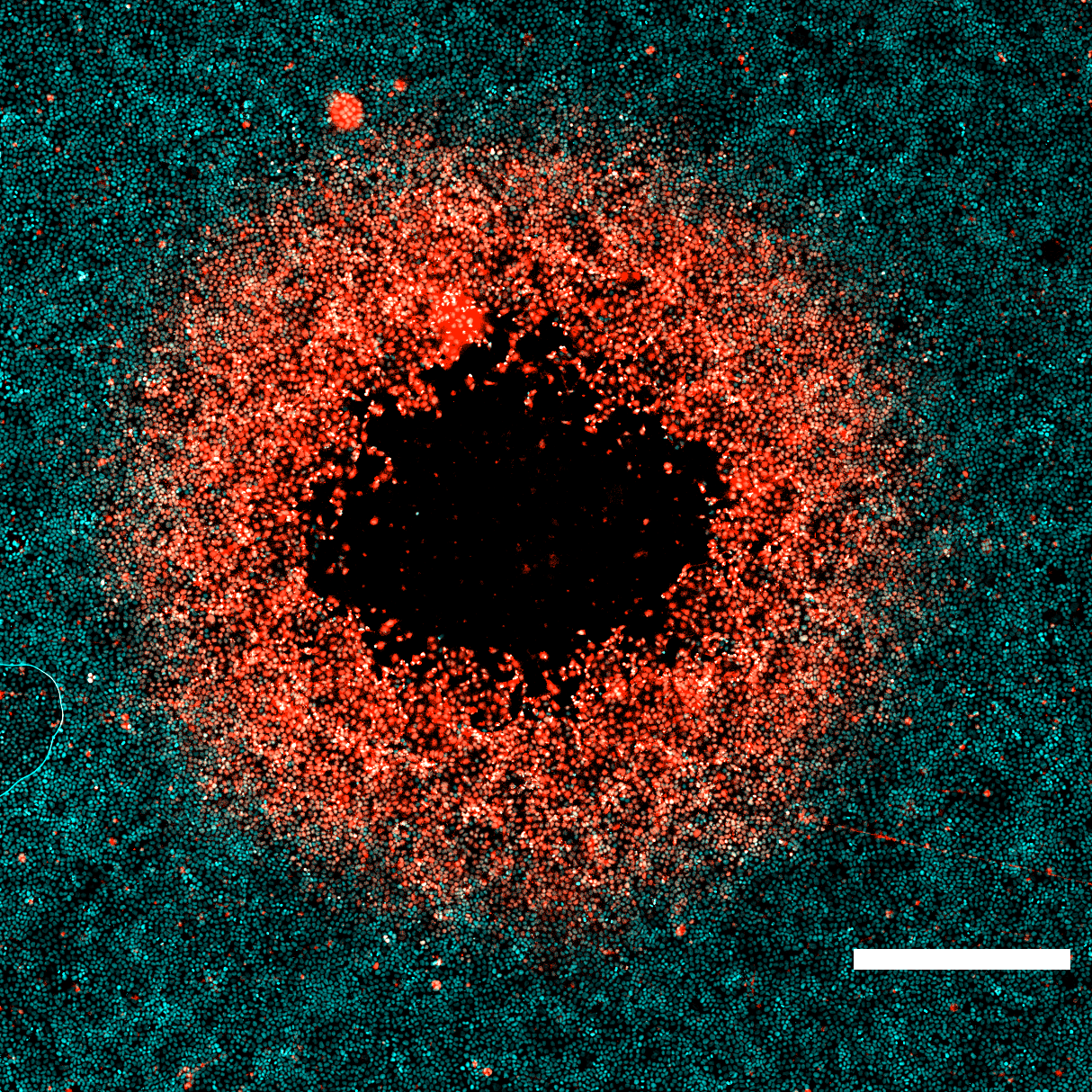

Supplement: Supplementary file 2 — Source data Fig. 1 [file 44318_2025_481_MOESM2_ESM.zip › Figure_01/1G/Fluorescent-plaque_36hpi_cropped.png]

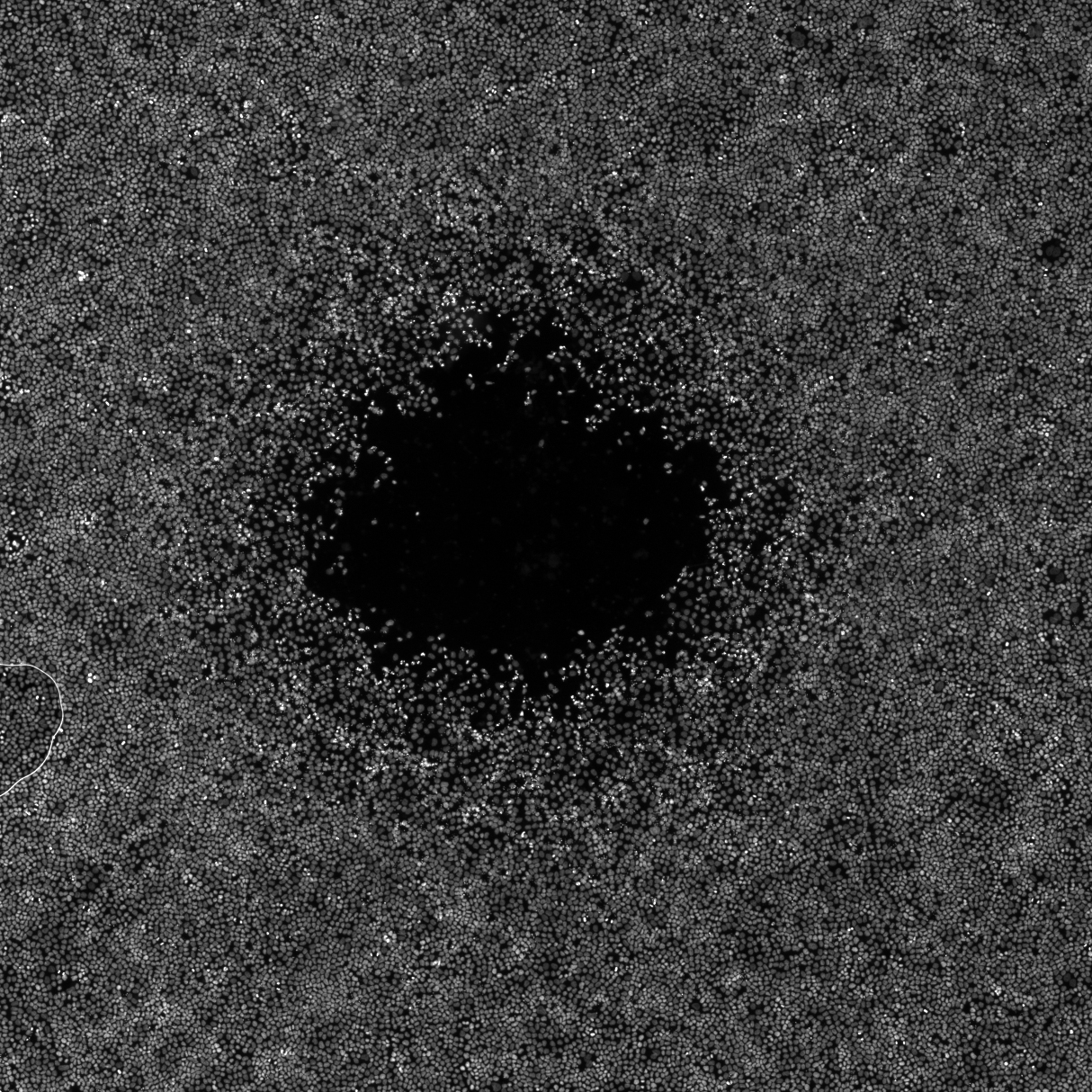

Supplement: Supplementary file 2 — Source data Fig. 1 [file 44318_2025_481_MOESM2_ESM.zip › Figure_01/1G/Fluorescent-plaque_36hpi_cropped.tif]

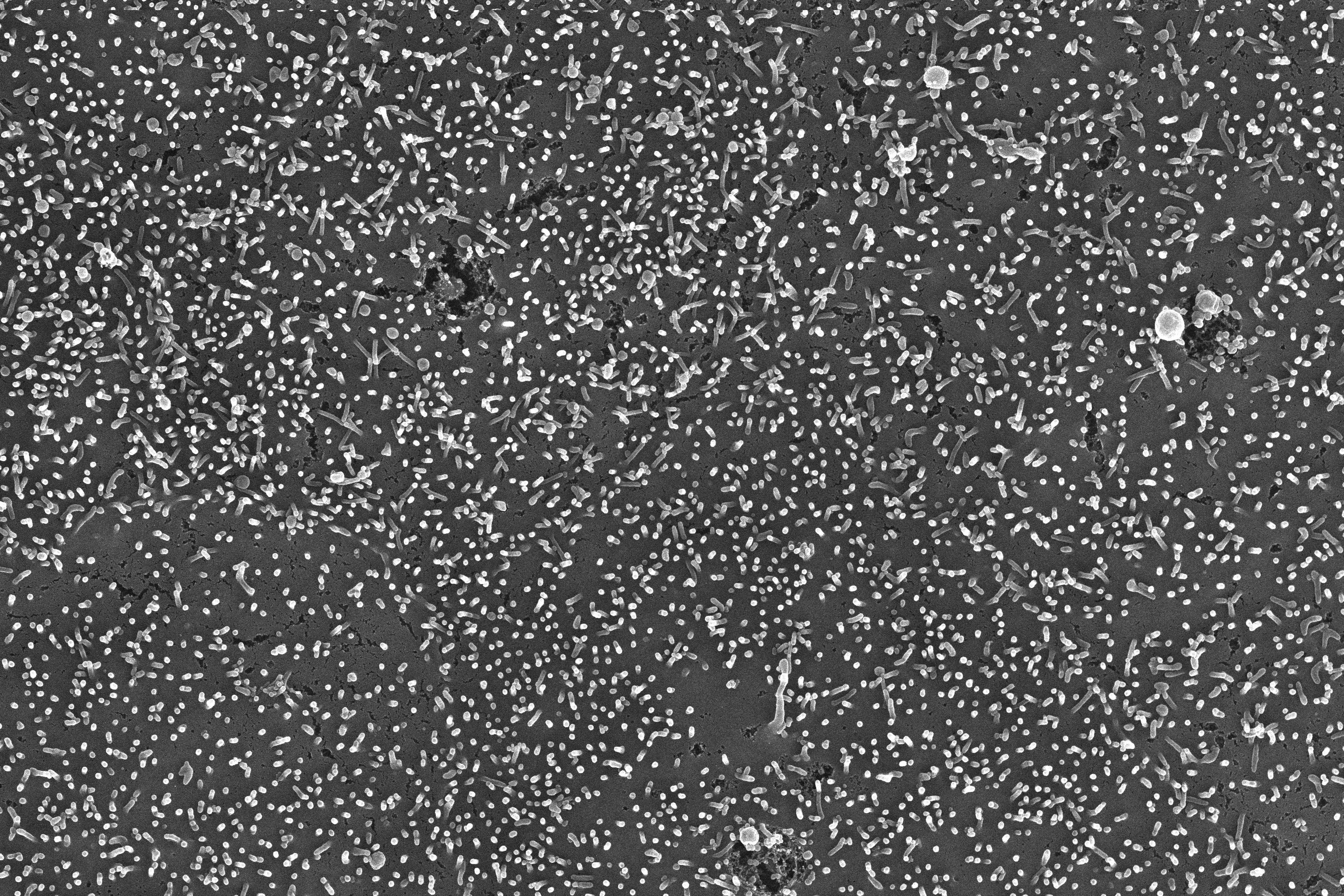

Supplement: Supplementary file 3 — Source data Fig. 2 [file 44318_2025_481_MOESM3_ESM.zip › Figure_02/2D/2D_01.tiff]

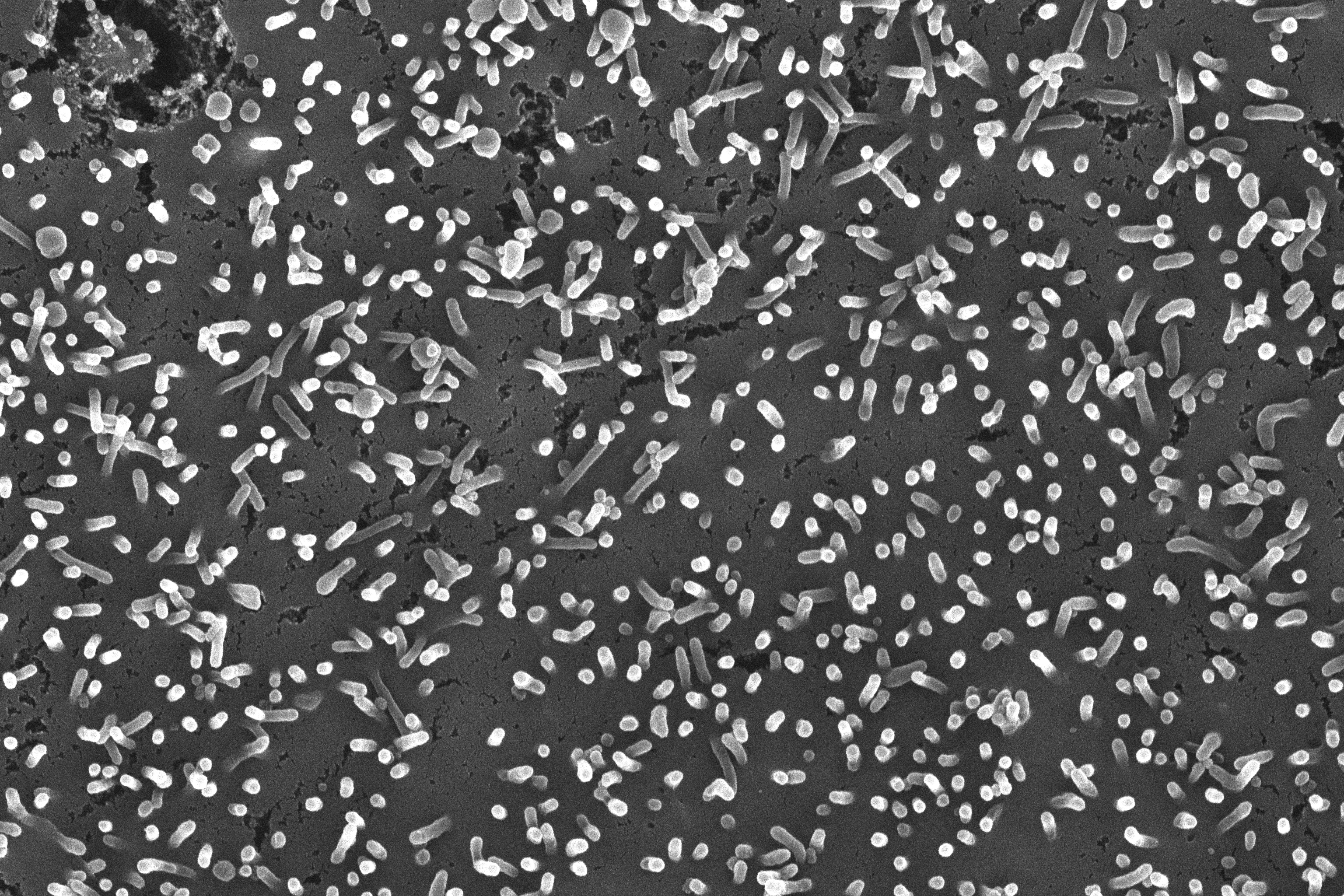

Supplement: Supplementary file 3 — Source data Fig. 2 [file 44318_2025_481_MOESM3_ESM.zip › Figure_02/2D/2D_02.tiff]

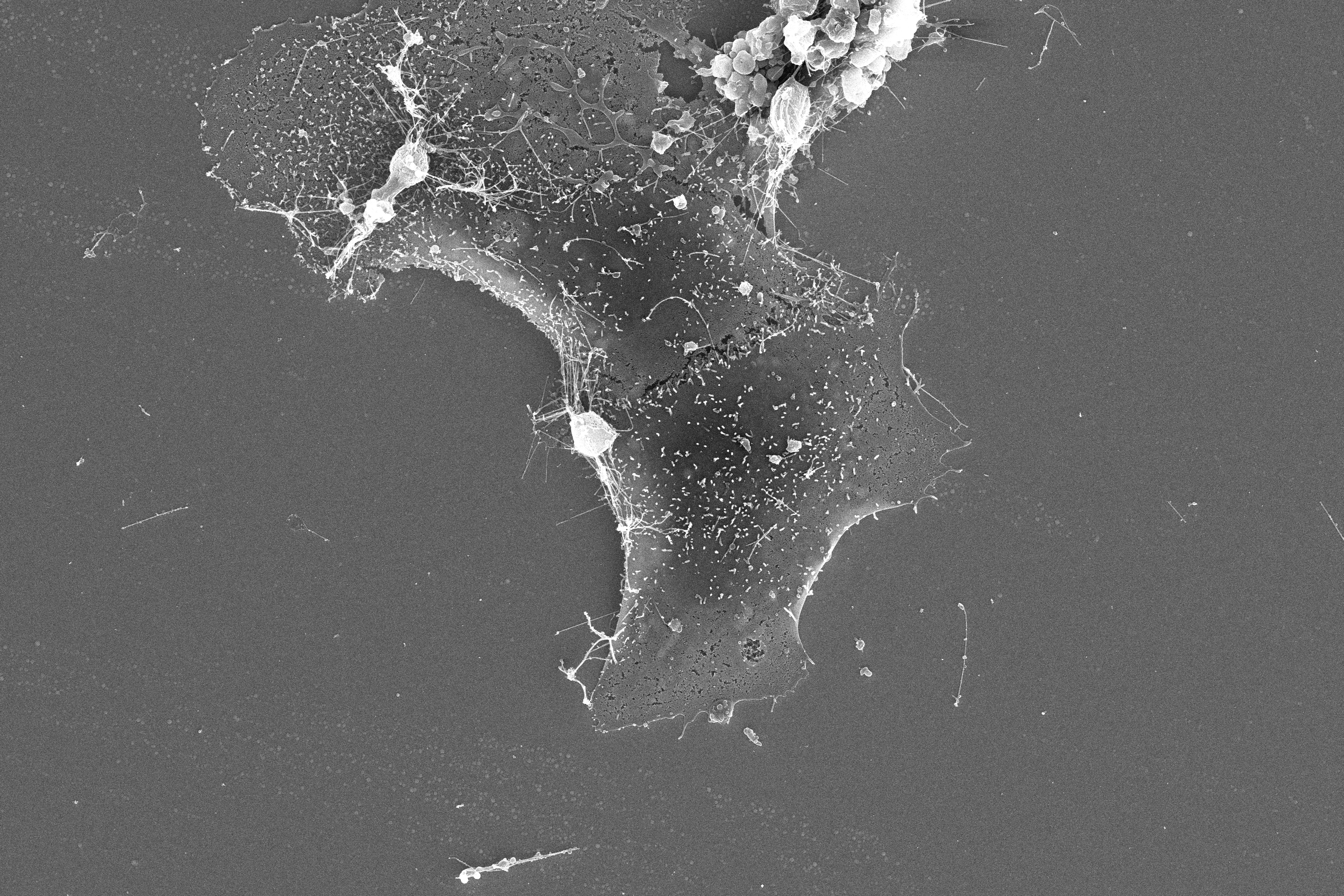

Supplement: Supplementary file 3 — Source data Fig. 2 [file 44318_2025_481_MOESM3_ESM.zip › Figure_02/2L/2L_01.tiff]

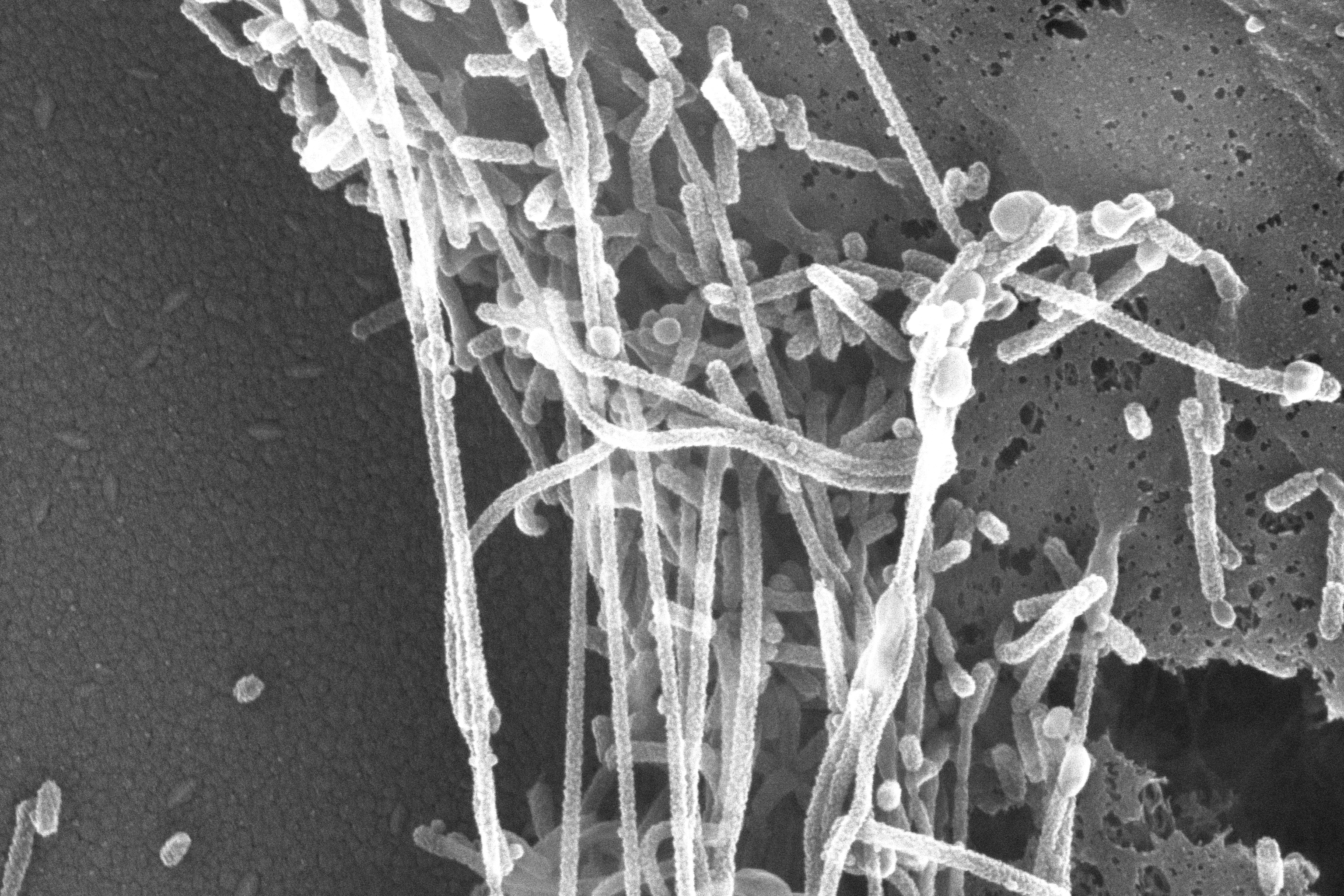

Supplement: Supplementary file 3 — Source data Fig. 2 [file 44318_2025_481_MOESM3_ESM.zip › Figure_02/2L/2L_03.tiff]

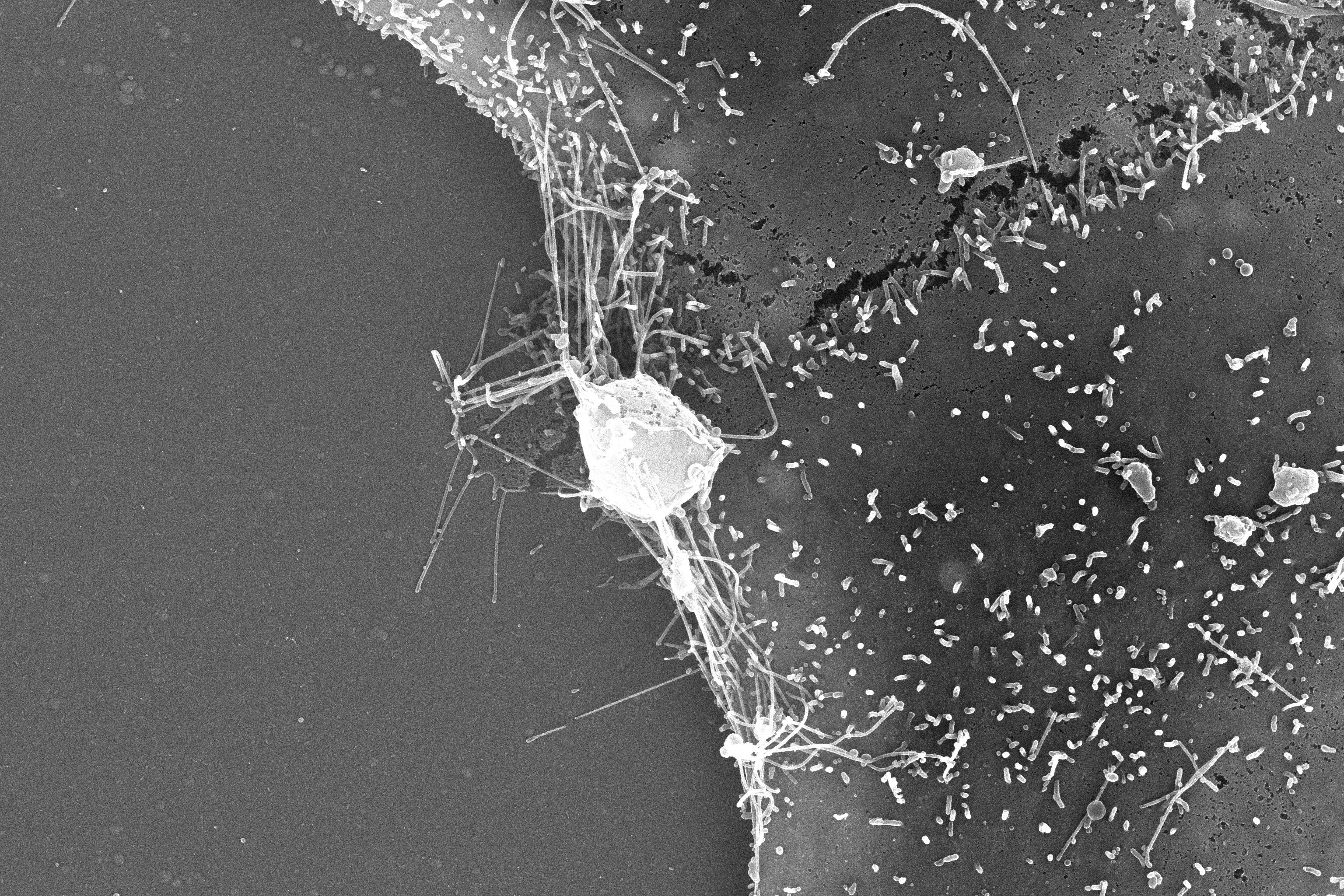

Supplement: Supplementary file 3 — Source data Fig. 2 [file 44318_2025_481_MOESM3_ESM.zip › Figure_02/2L/2L_02.tiff]

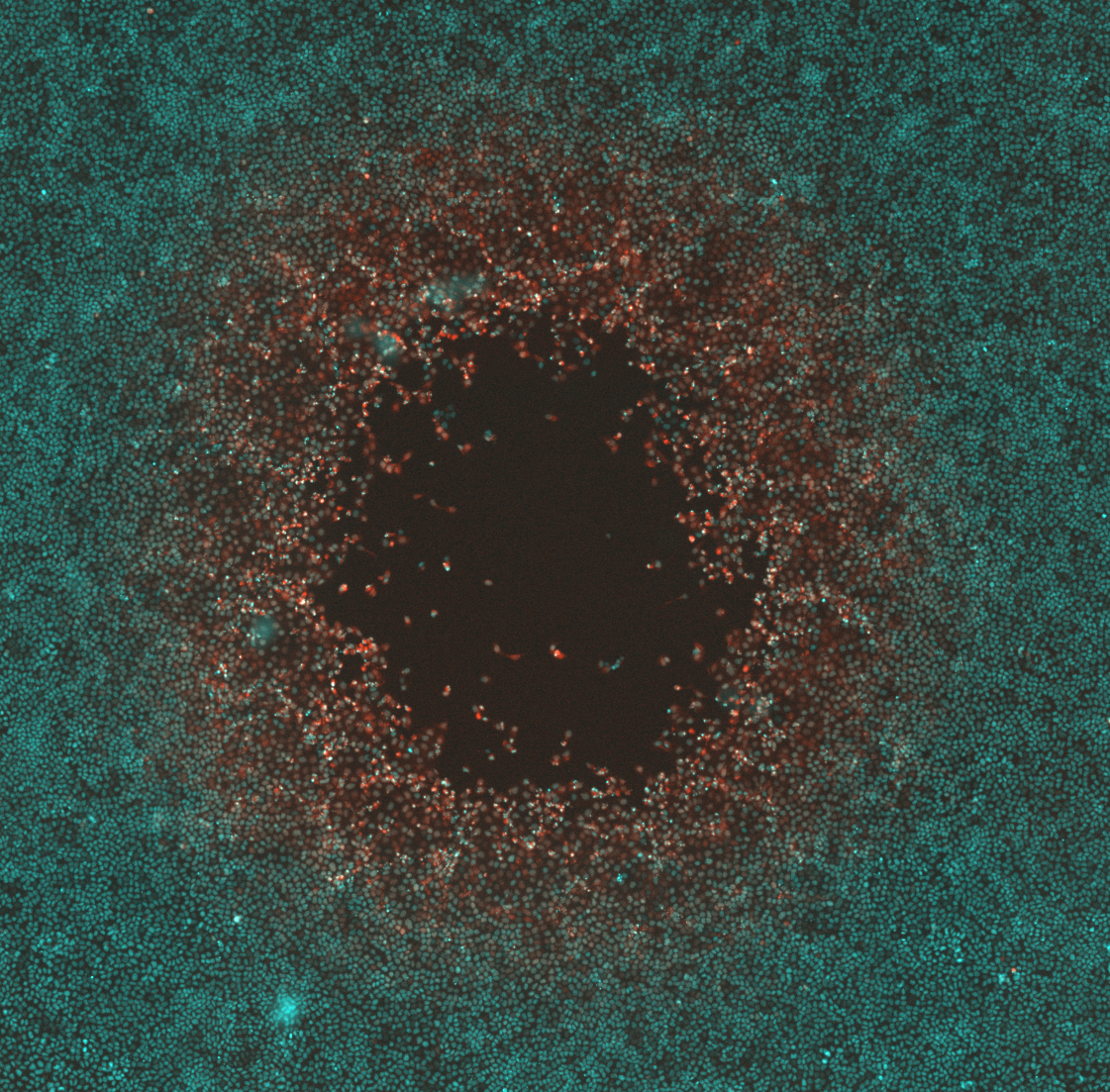

Supplement: Supplementary file 3 — Source data Fig. 2 [file 44318_2025_481_MOESM3_ESM.zip › Figure_02/2G/WSN-M1-Udorn-PAmScarlet_42hpi-zoom.tif]

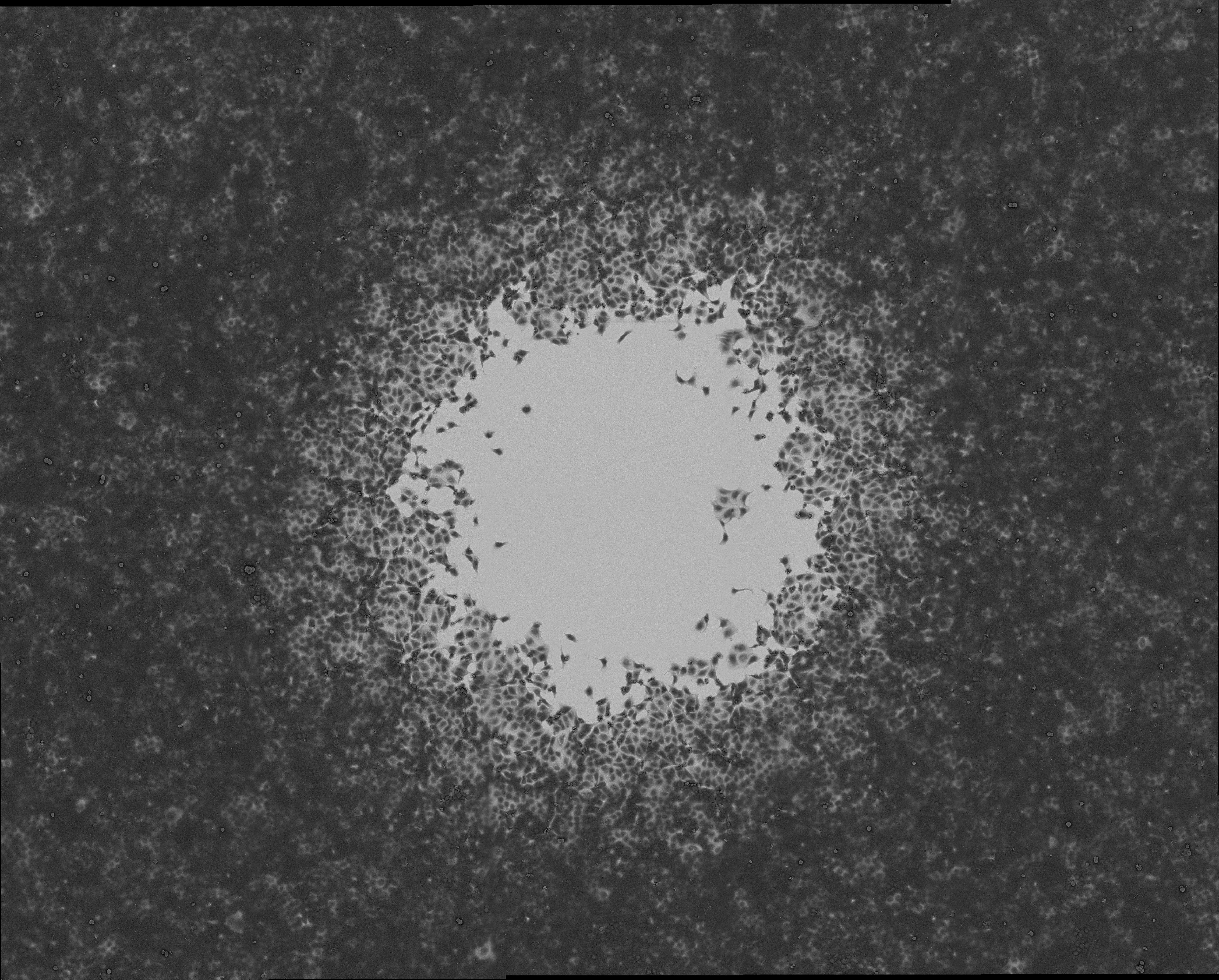

Supplement: Supplementary file 3 — Source data Fig. 2 [file 44318_2025_481_MOESM3_ESM.zip › Figure_02/2B/SEM_WSN-PAmScarlet_36hpi.tif]

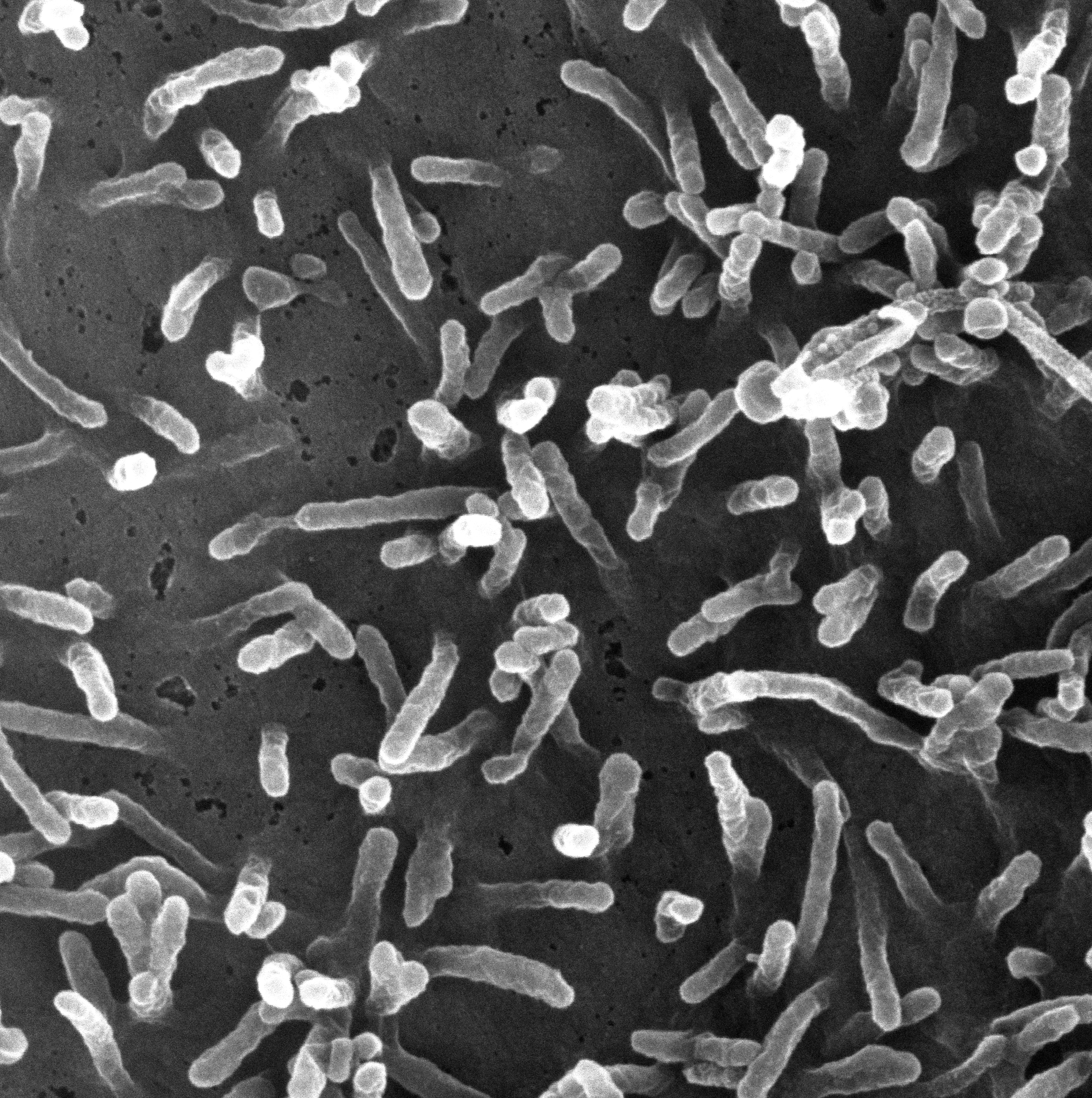

Supplement: Supplementary file 3 — Source data Fig. 2 [file 44318_2025_481_MOESM3_ESM.zip › Figure_02/2J/2J_02.tif]

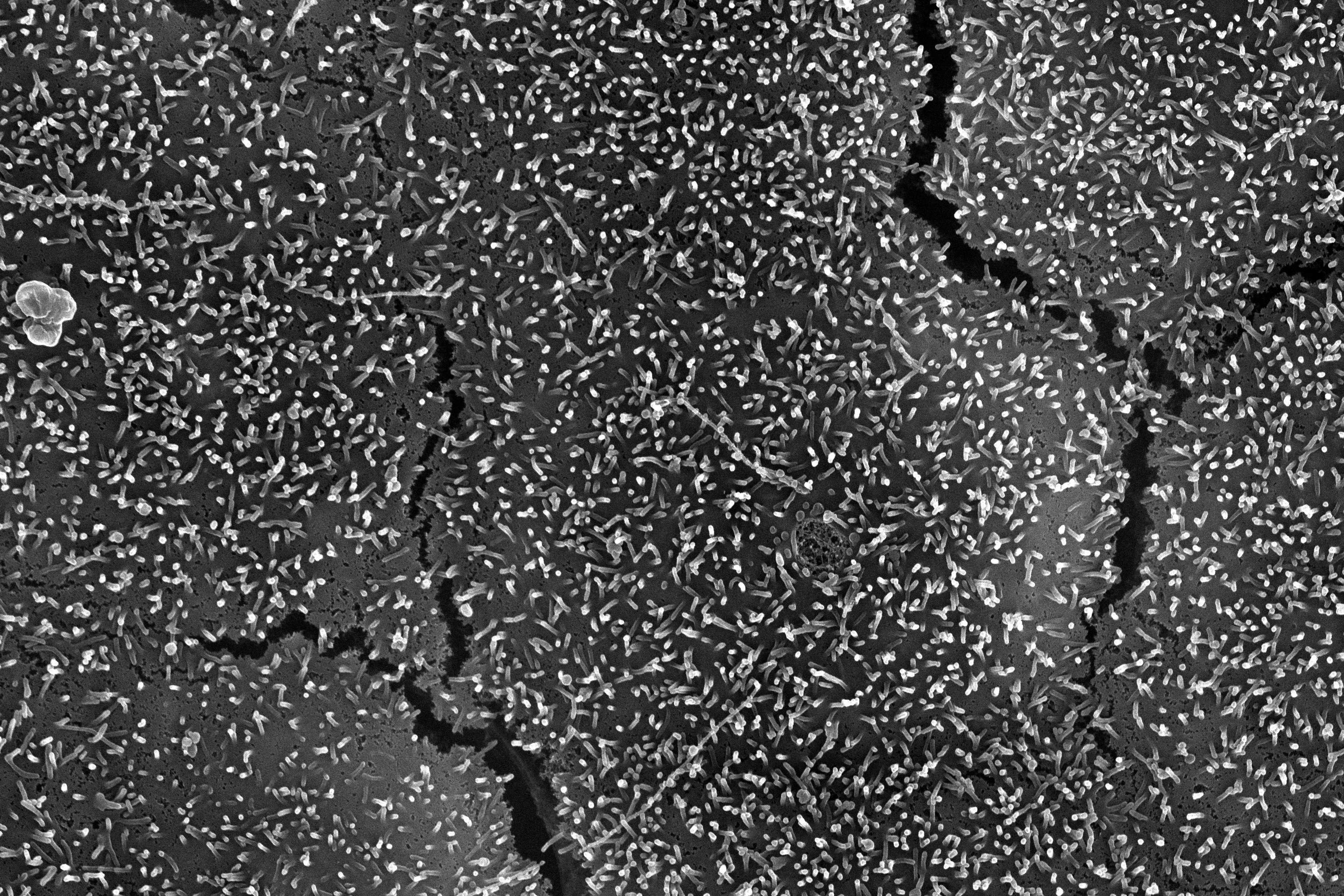

Supplement: Supplementary file 3 — Source data Fig. 2 [file 44318_2025_481_MOESM3_ESM.zip › Figure_02/2J/2J_01.tiff]

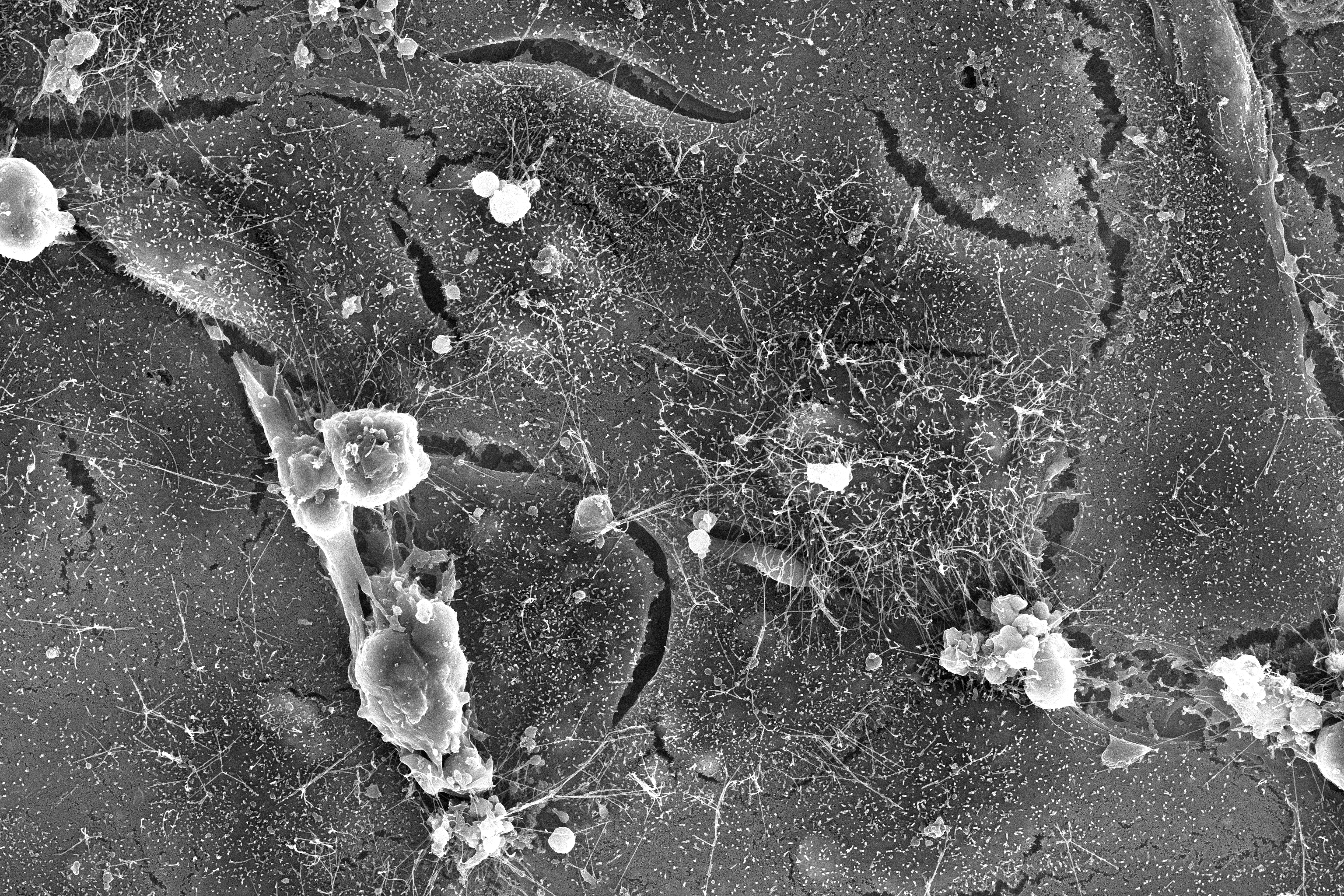

Supplement: Supplementary file 3 — Source data Fig. 2 [file 44318_2025_481_MOESM3_ESM.zip › Figure_02/2K/2K_01.tiff]

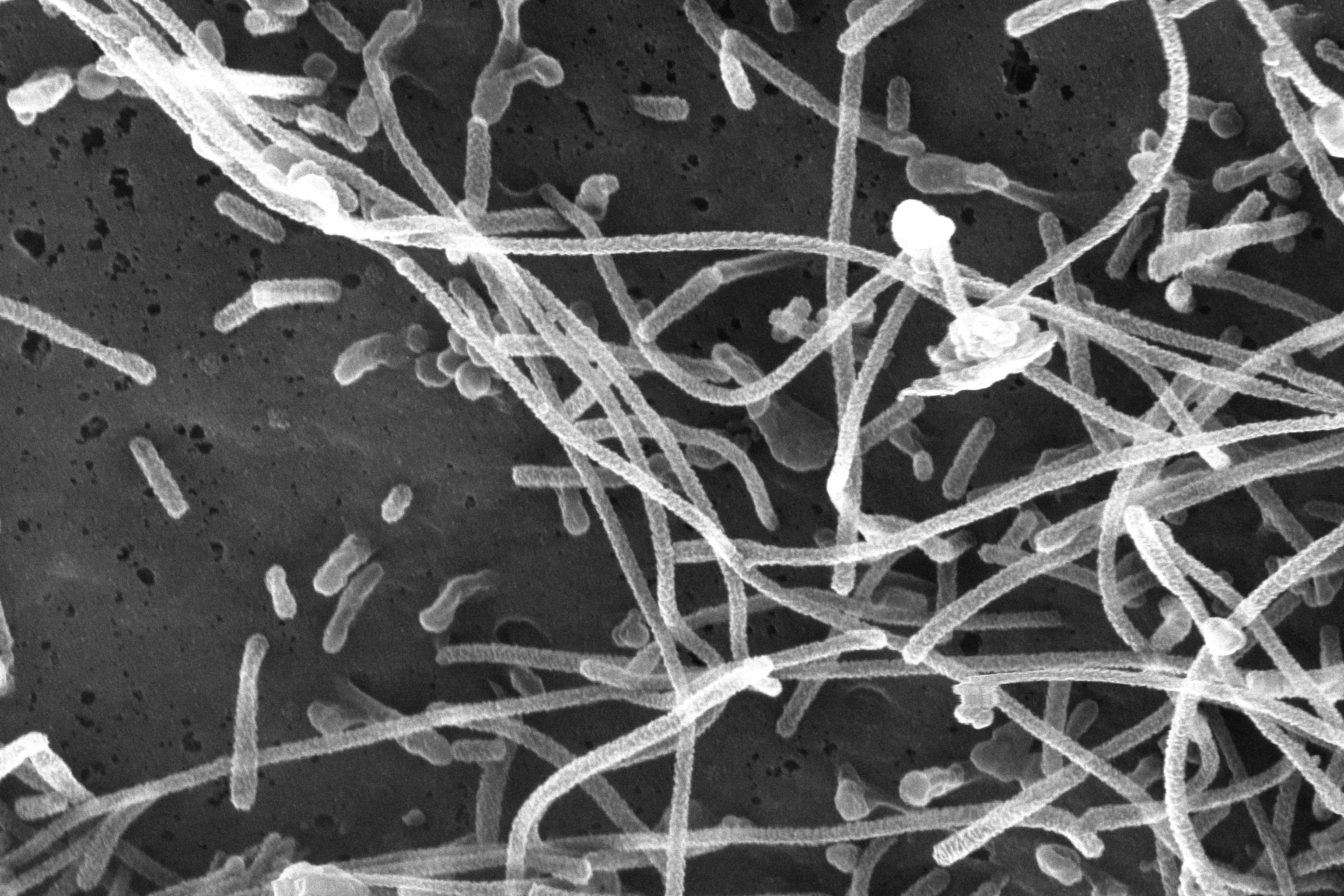

Supplement: Supplementary file 3 — Source data Fig. 2 [file 44318_2025_481_MOESM3_ESM.zip › Figure_02/2K/2K_03.tiff]

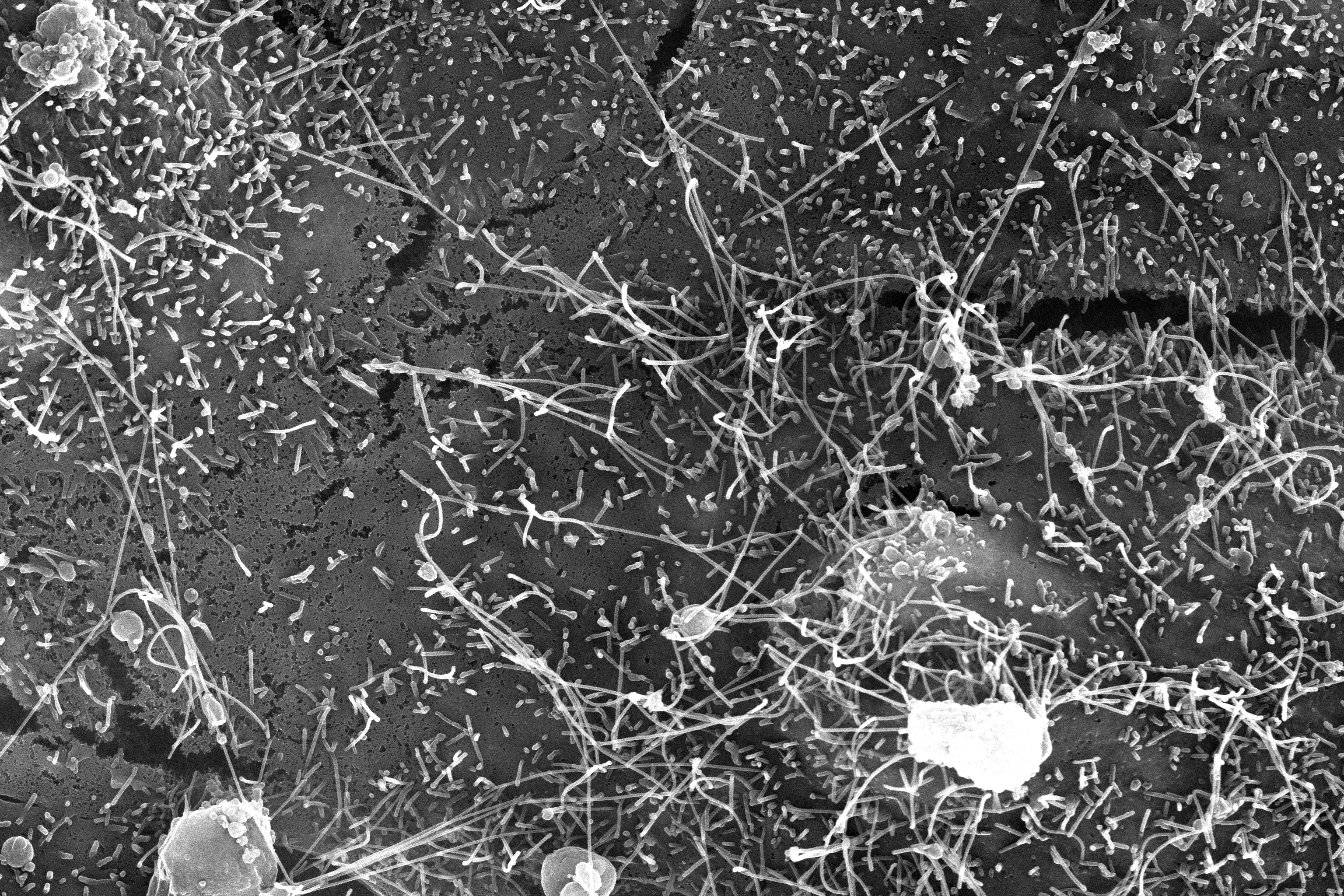

Supplement: Supplementary file 3 — Source data Fig. 2 [file 44318_2025_481_MOESM3_ESM.zip › Figure_02/2K/2K_02.tiff]

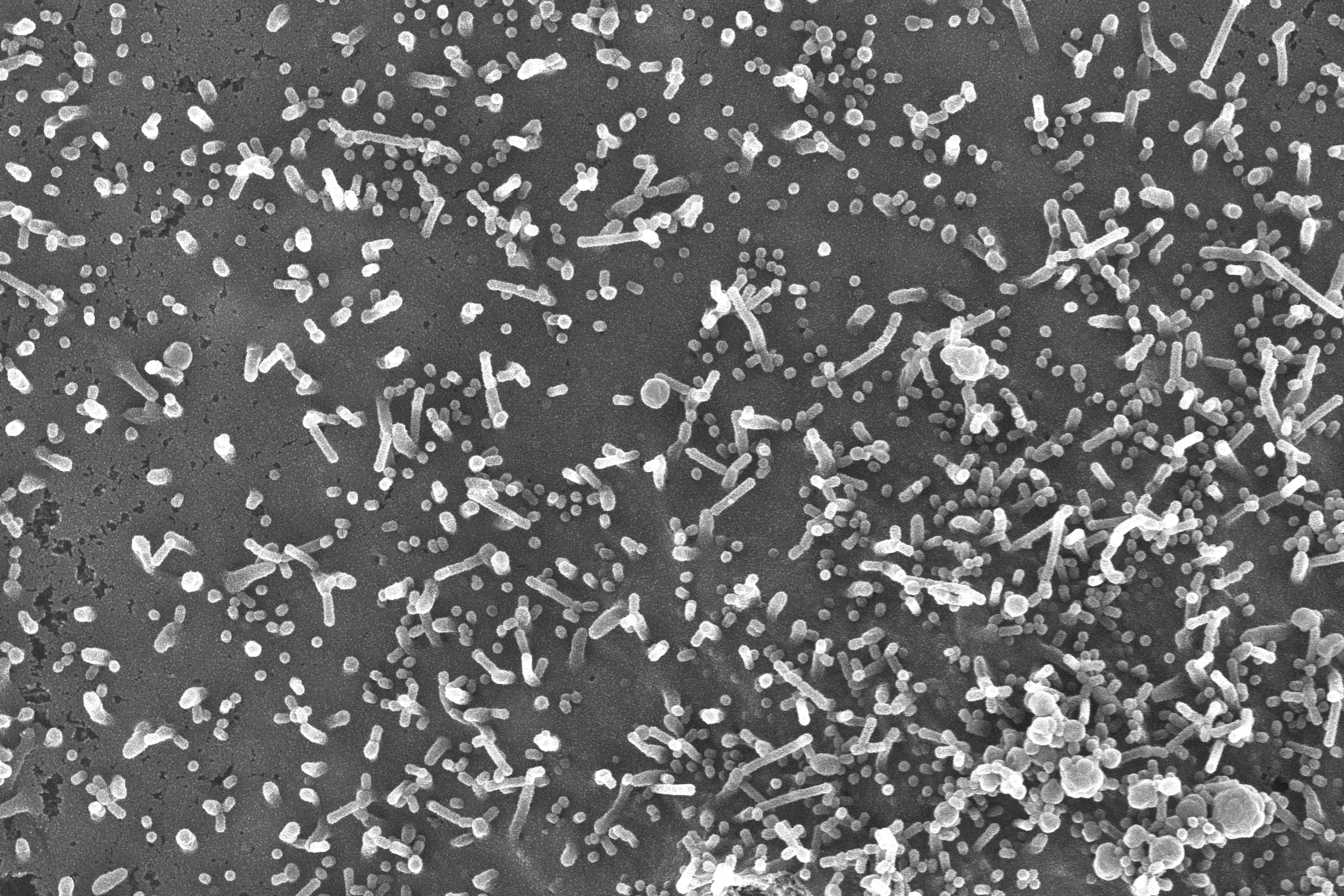

Supplement: Supplementary file 3 — Source data Fig. 2 [file 44318_2025_481_MOESM3_ESM.zip › Figure_02/2F/2F_02.tiff]

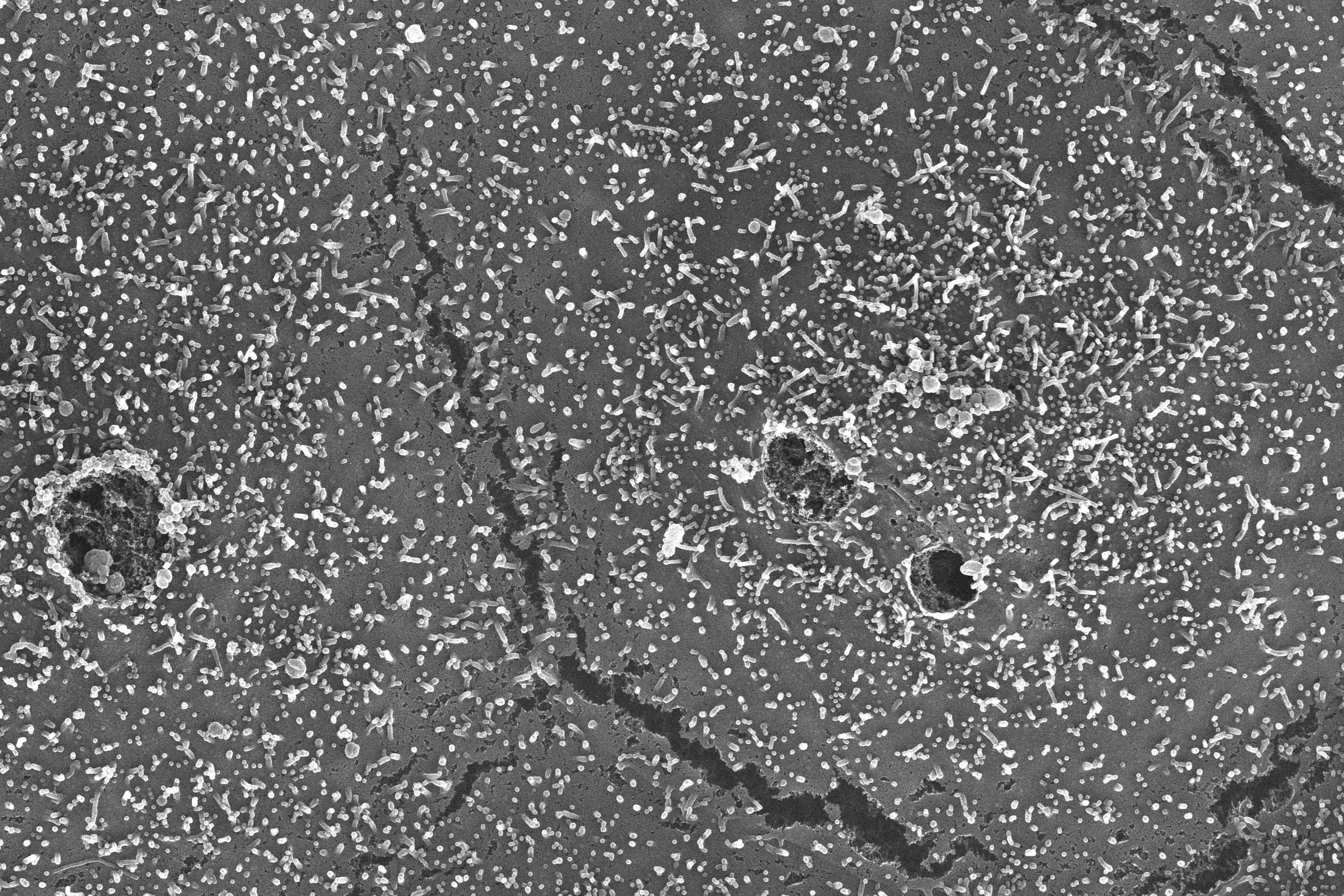

Supplement: Supplementary file 3 — Source data Fig. 2 [file 44318_2025_481_MOESM3_ESM.zip › Figure_02/2F/2F_01.tiff]

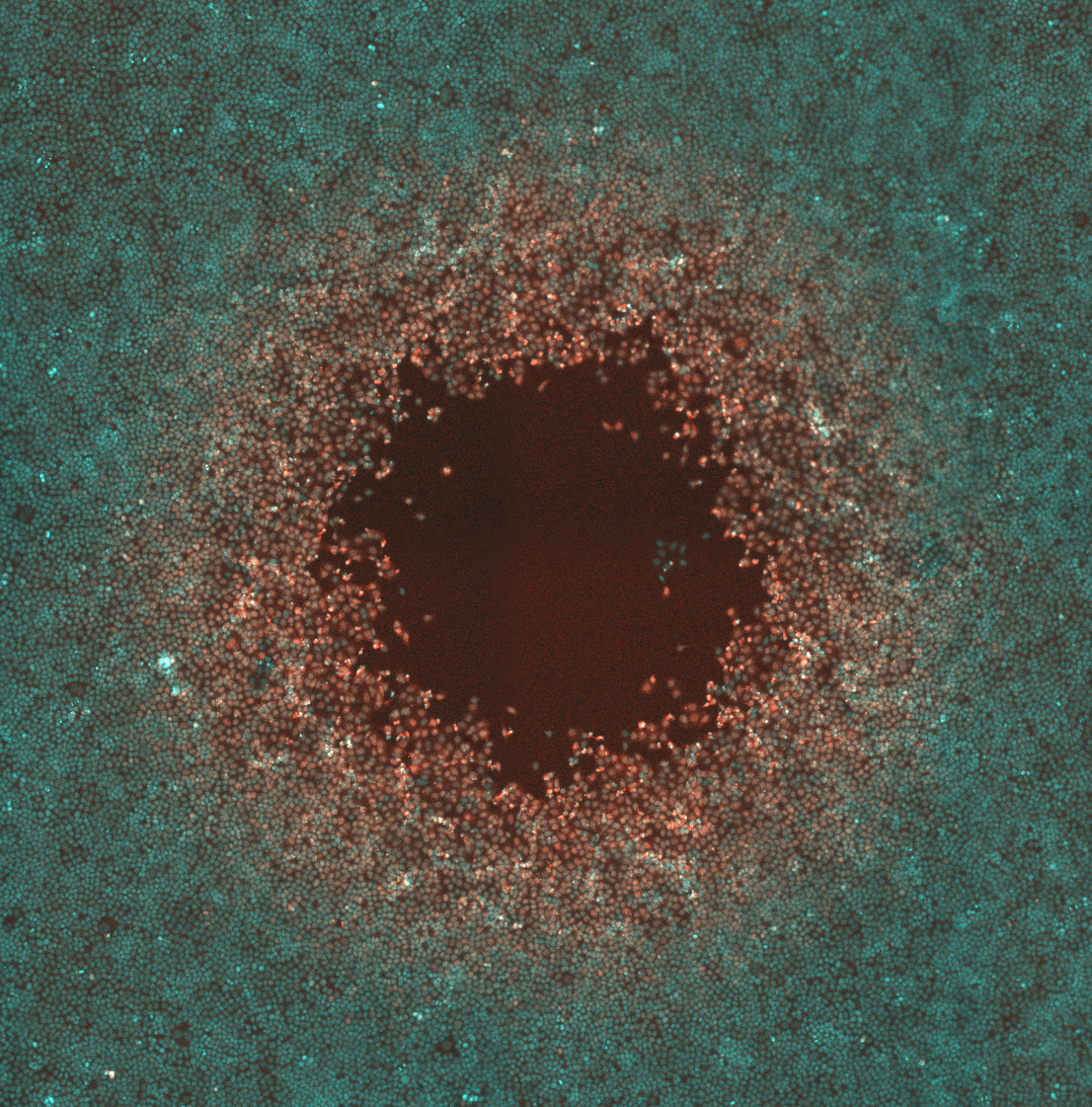

Supplement: Supplementary file 3 — Source data Fig. 2 [file 44318_2025_481_MOESM3_ESM.zip › Figure_02/2A/WSN-PAmScarlet_36hpi-zoom.tif]

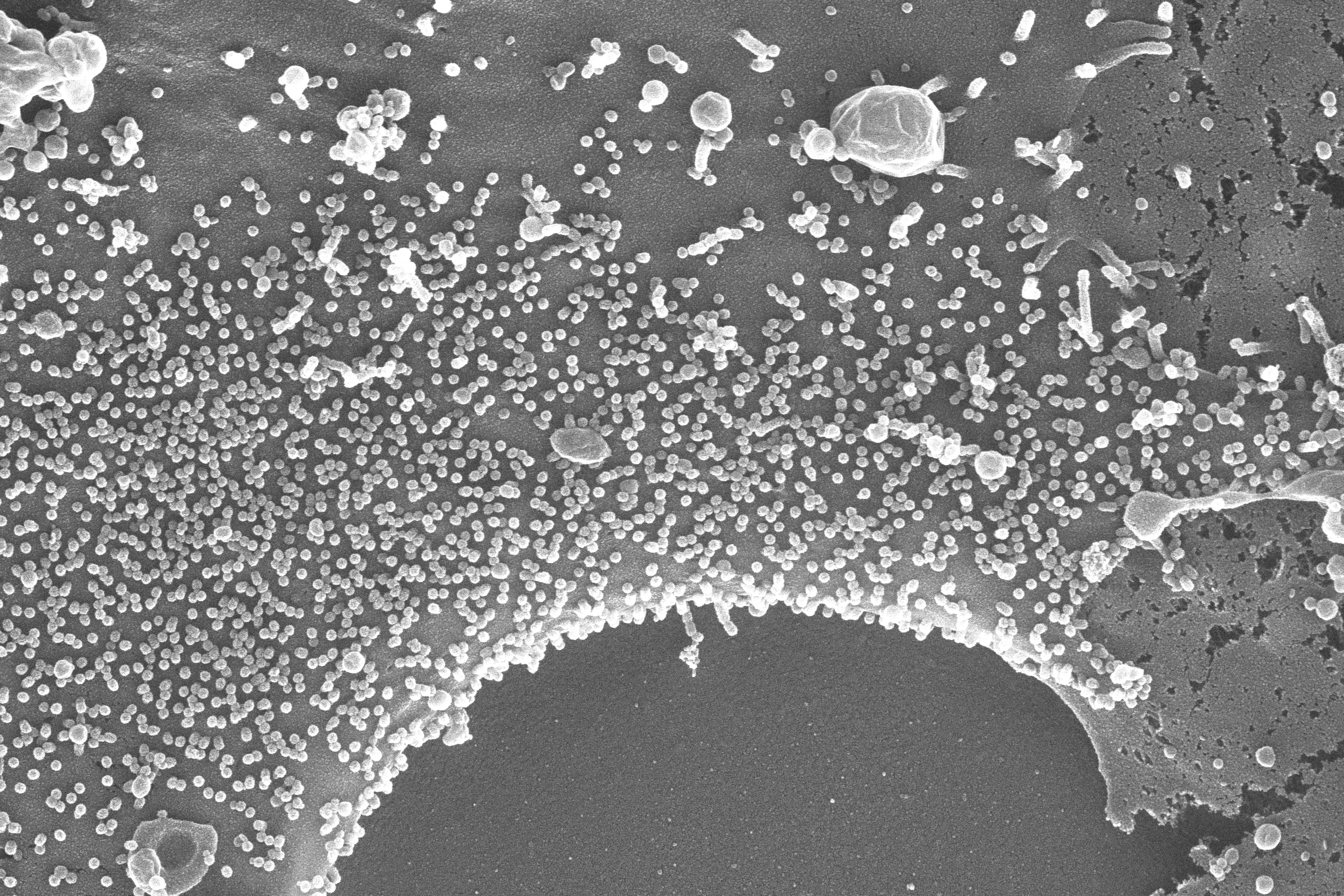

Supplement: Supplementary file 3 — Source data Fig. 2 [file 44318_2025_481_MOESM3_ESM.zip › Figure_02/2E/2E_03.tiff]

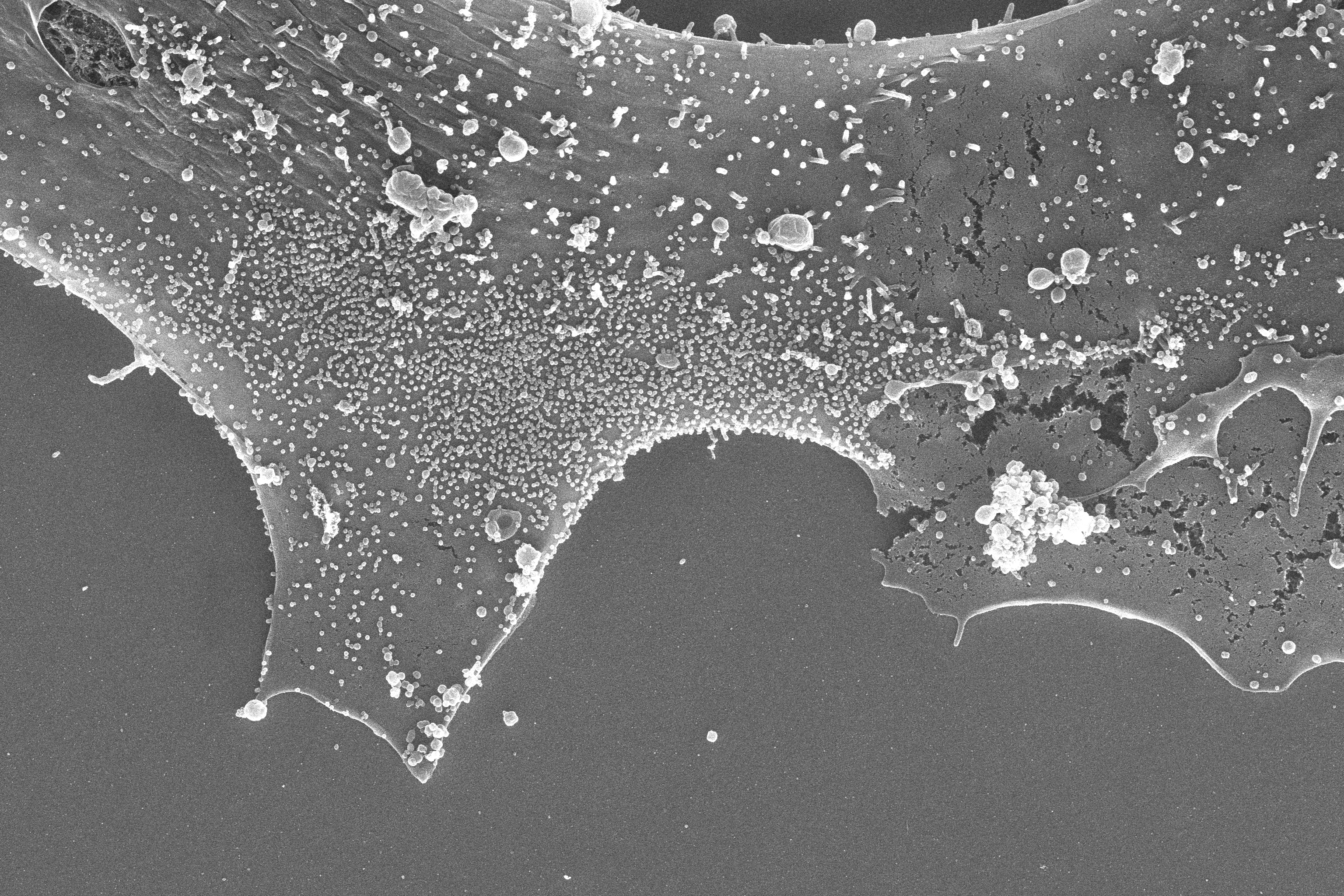

Supplement: Supplementary file 3 — Source data Fig. 2 [file 44318_2025_481_MOESM3_ESM.zip › Figure_02/2E/2E_02.tiff]

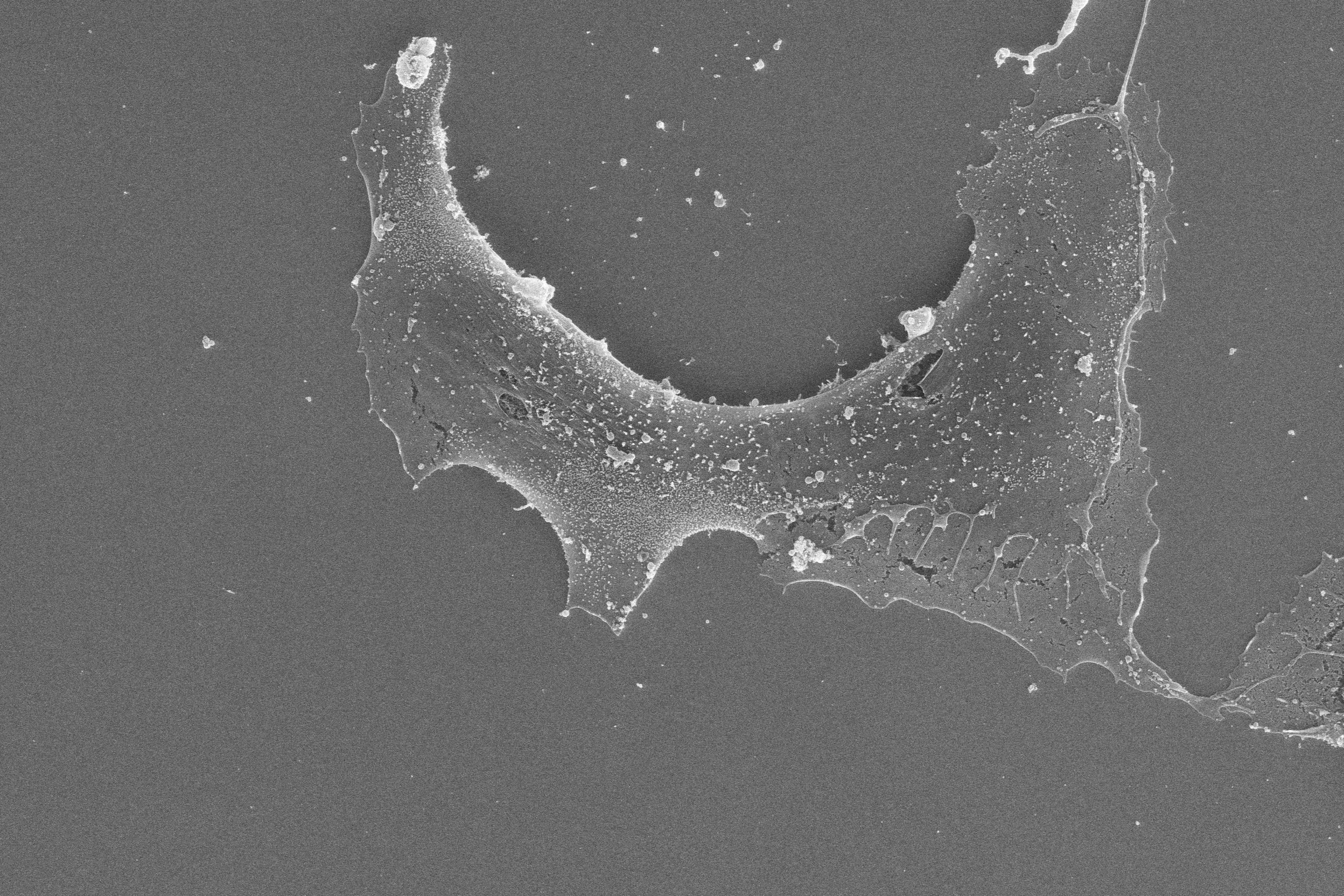

Supplement: Supplementary file 3 — Source data Fig. 2 [file 44318_2025_481_MOESM3_ESM.zip › Figure_02/2E/2E_01.tiff]

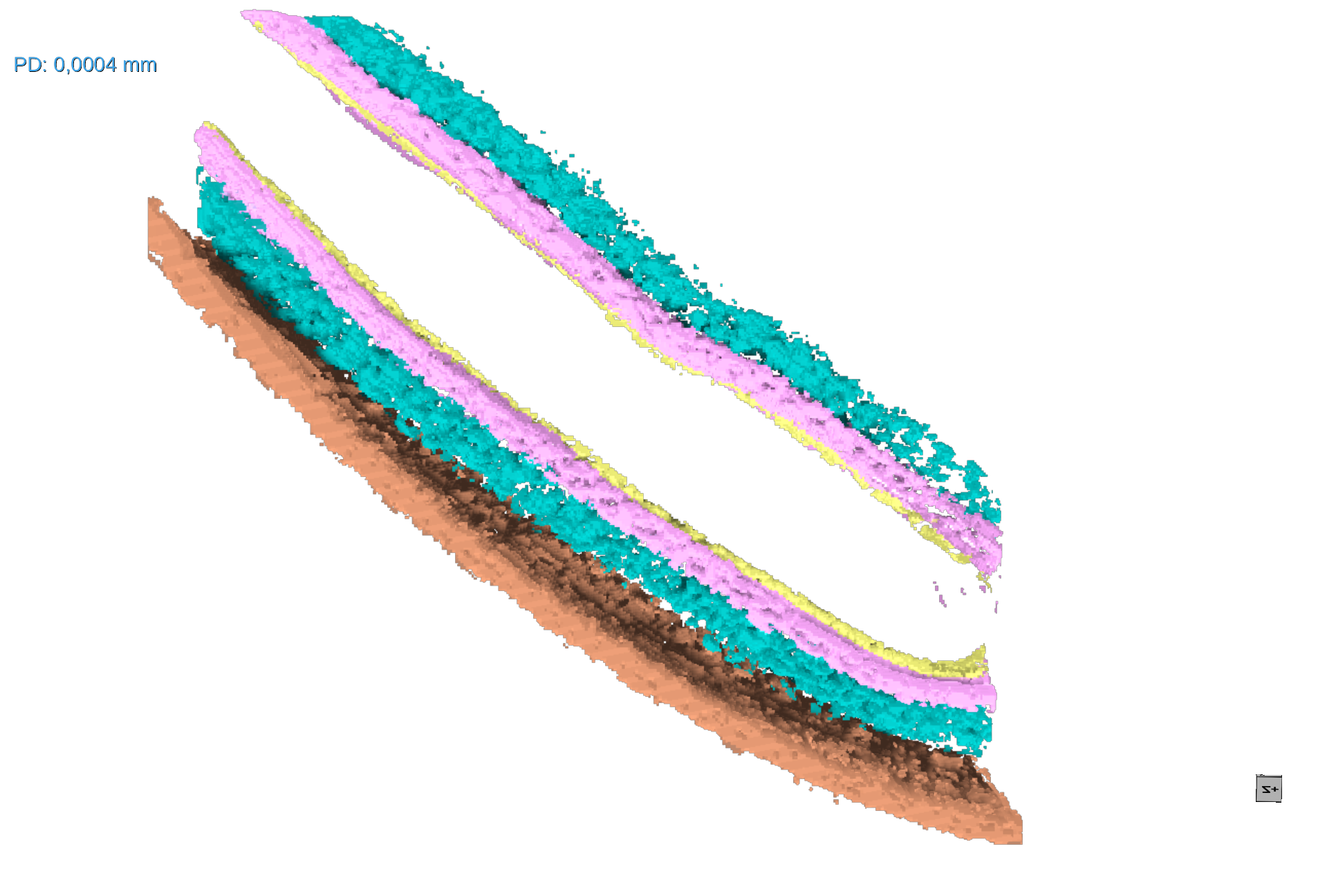

Supplement: Supplementary file 4 — Source data Fig. 3 [file 44318_2025_481_MOESM4_ESM.zip › Figure_03/3L/3L_WSN-M1-Udorn-zoom_Dragonfly.png]

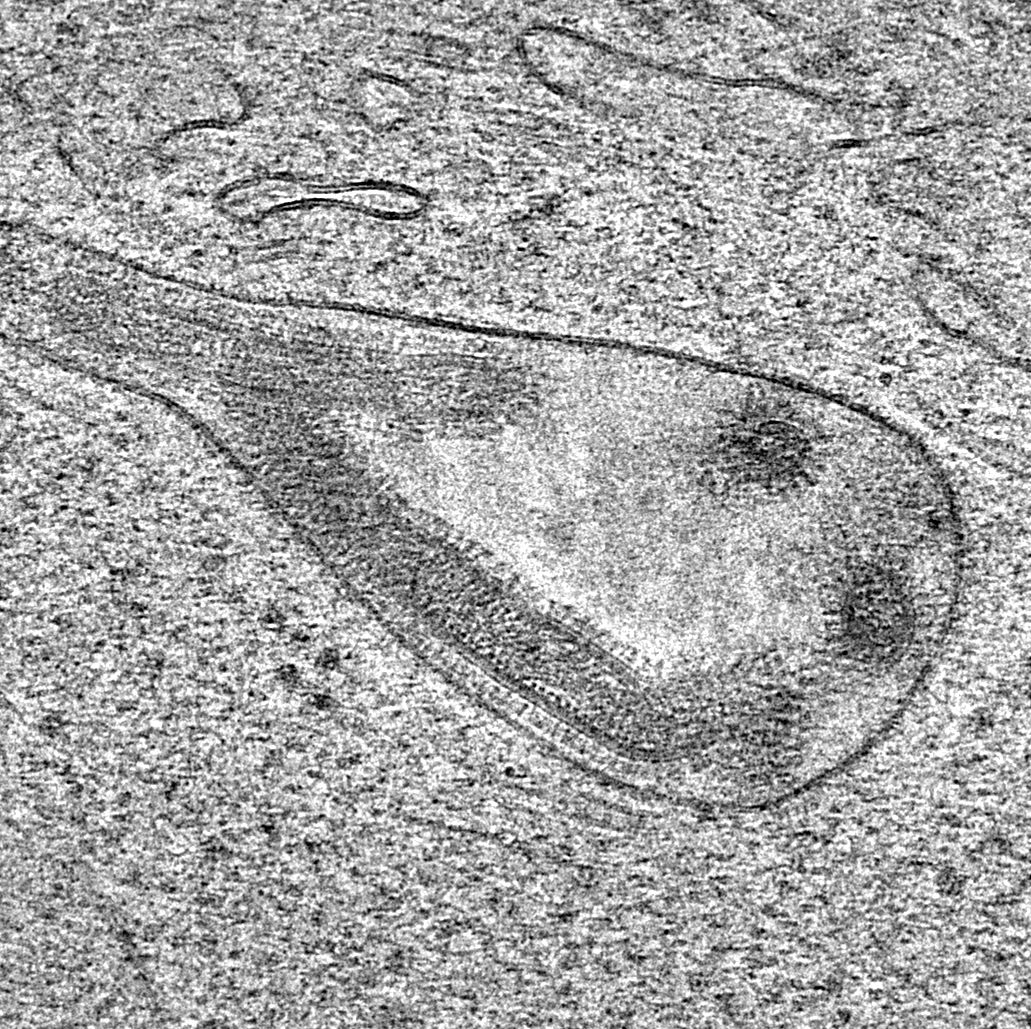

Supplement: Supplementary file 4 — Source data Fig. 3 [file 44318_2025_481_MOESM4_ESM.zip › Figure_03/3G/3G_WSN-M1-Udorn.jpg]

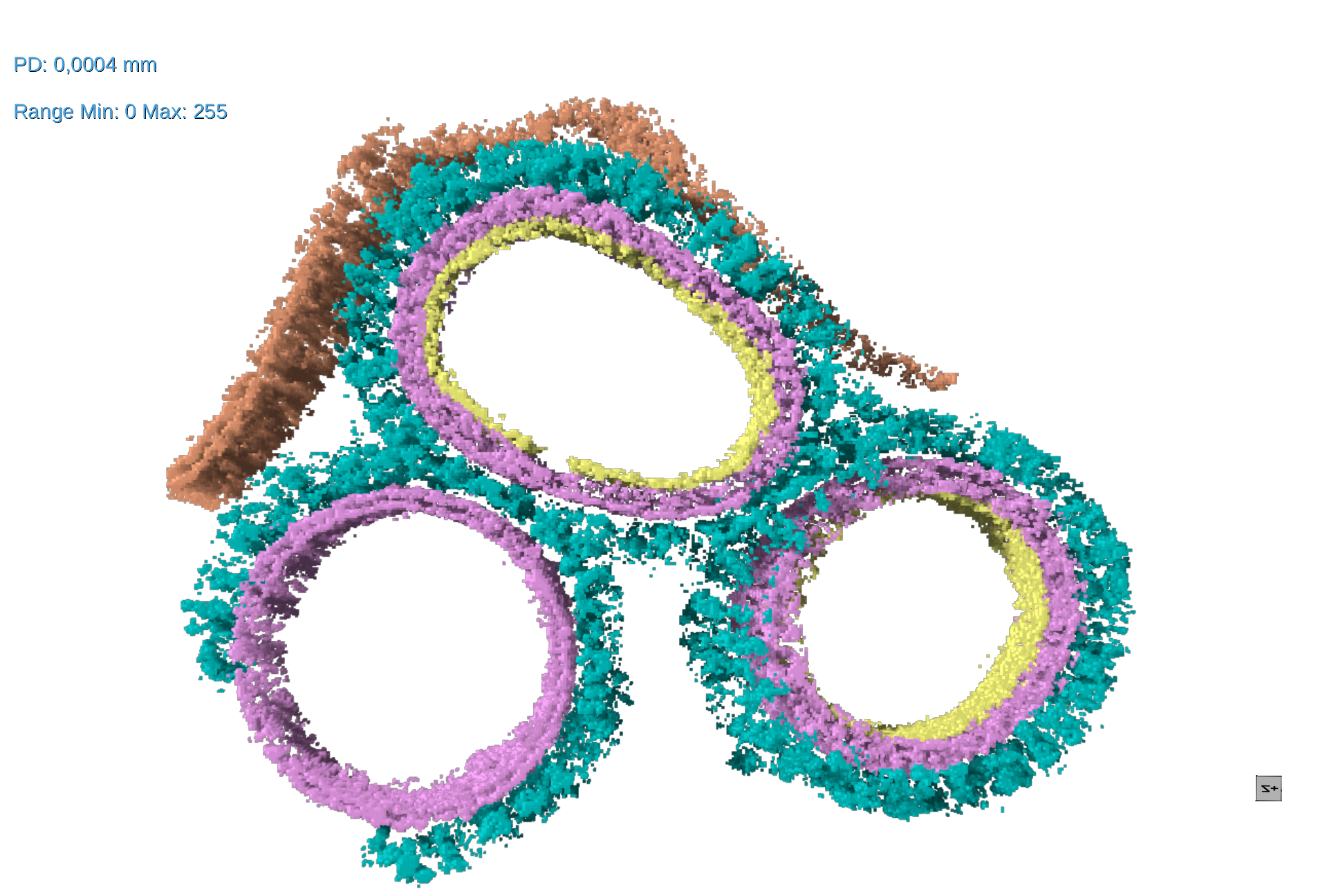

Supplement: Supplementary file 4 — Source data Fig. 3 [file 44318_2025_481_MOESM4_ESM.zip › Figure_03/3J/3J_WSN-zoom_Dragonfly.png]

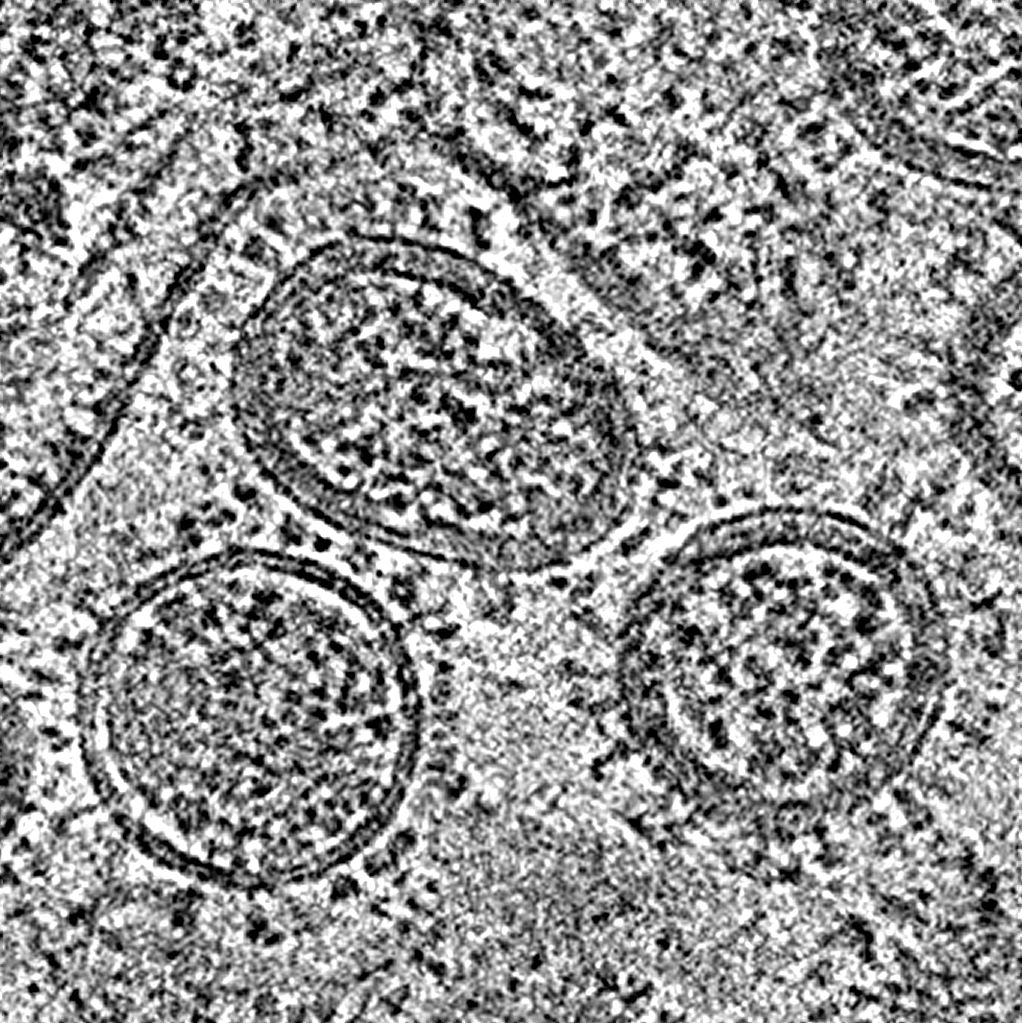

Supplement: Supplementary file 4 — Source data Fig. 3 [file 44318_2025_481_MOESM4_ESM.zip › Figure_03/3F/3F_WSN.jpg]

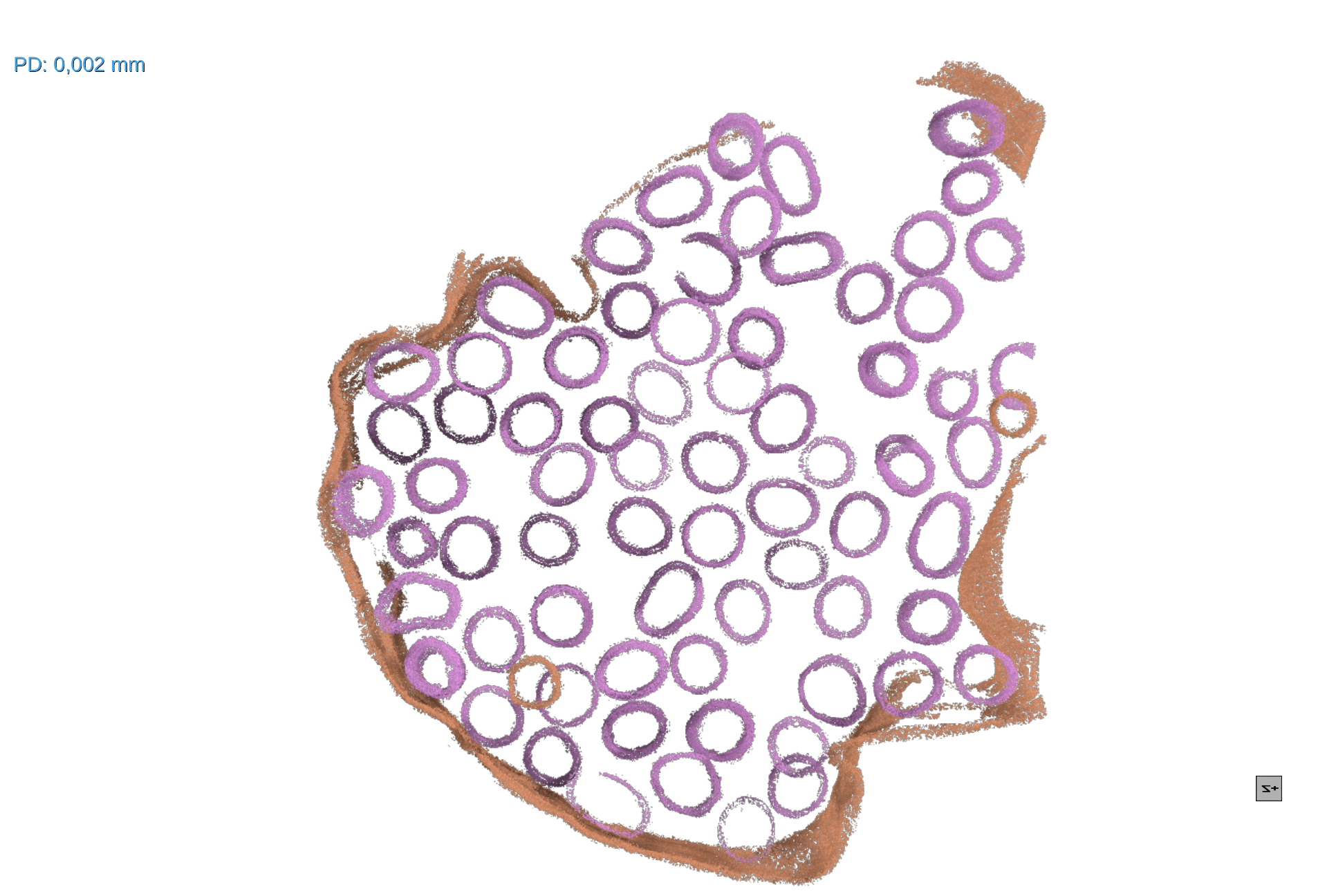

Supplement: Supplementary file 4 — Source data Fig. 3 [file 44318_2025_481_MOESM4_ESM.zip › Figure_03/3I/3I_WSN_Dragonfly.png]

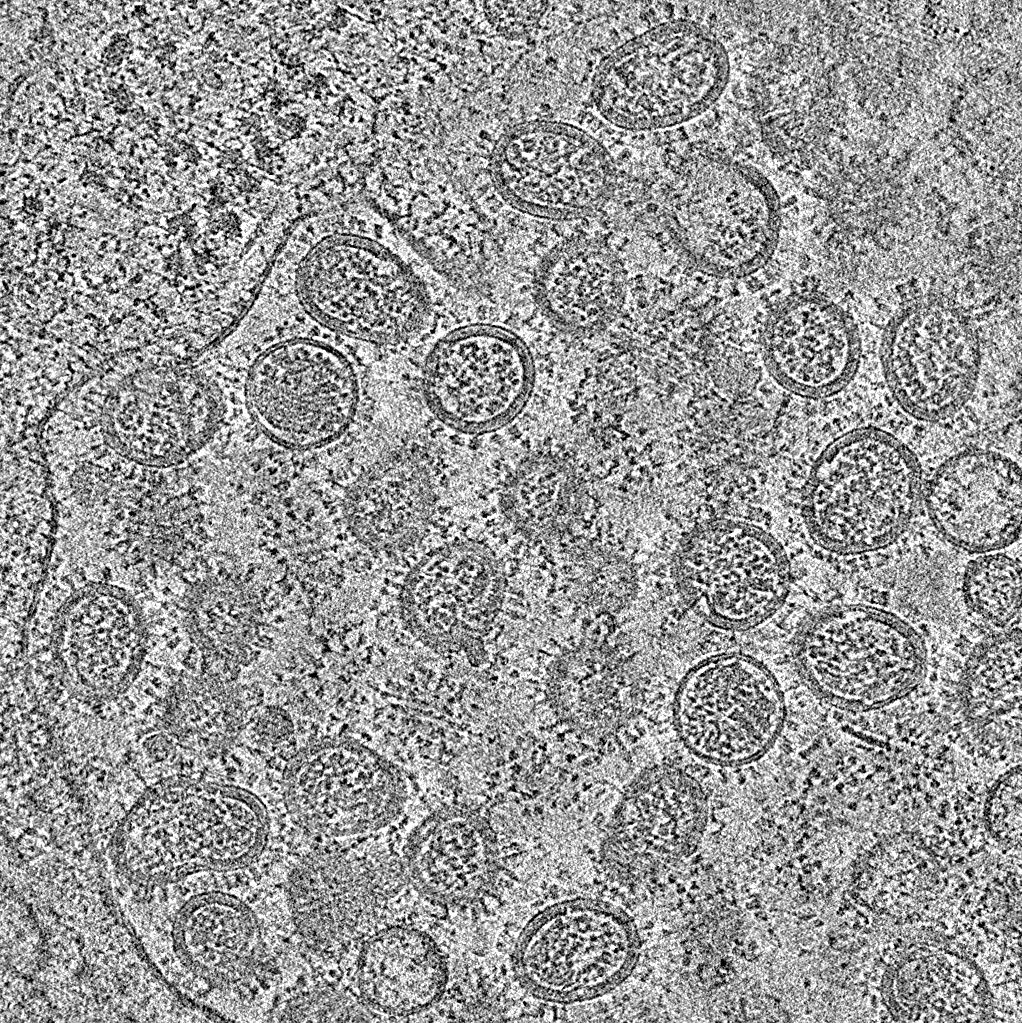

Supplement: Supplementary file 4 — Source data Fig. 3 [file 44318_2025_481_MOESM4_ESM.zip › Figure_03/3E/3E_WSN.jpg]

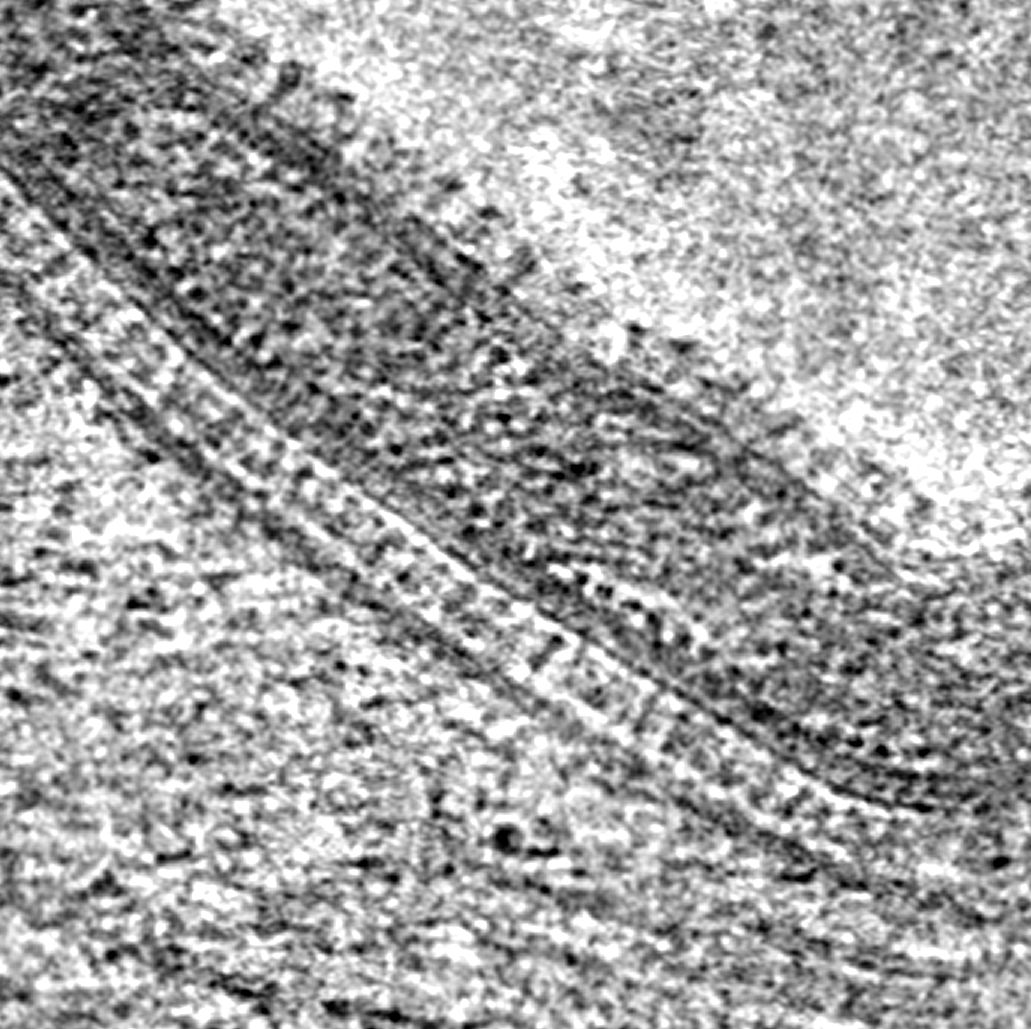

Supplement: Supplementary file 4 — Source data Fig. 3 [file 44318_2025_481_MOESM4_ESM.zip › Figure_03/3H/3H_WSN-M1-Udorn.jpg]

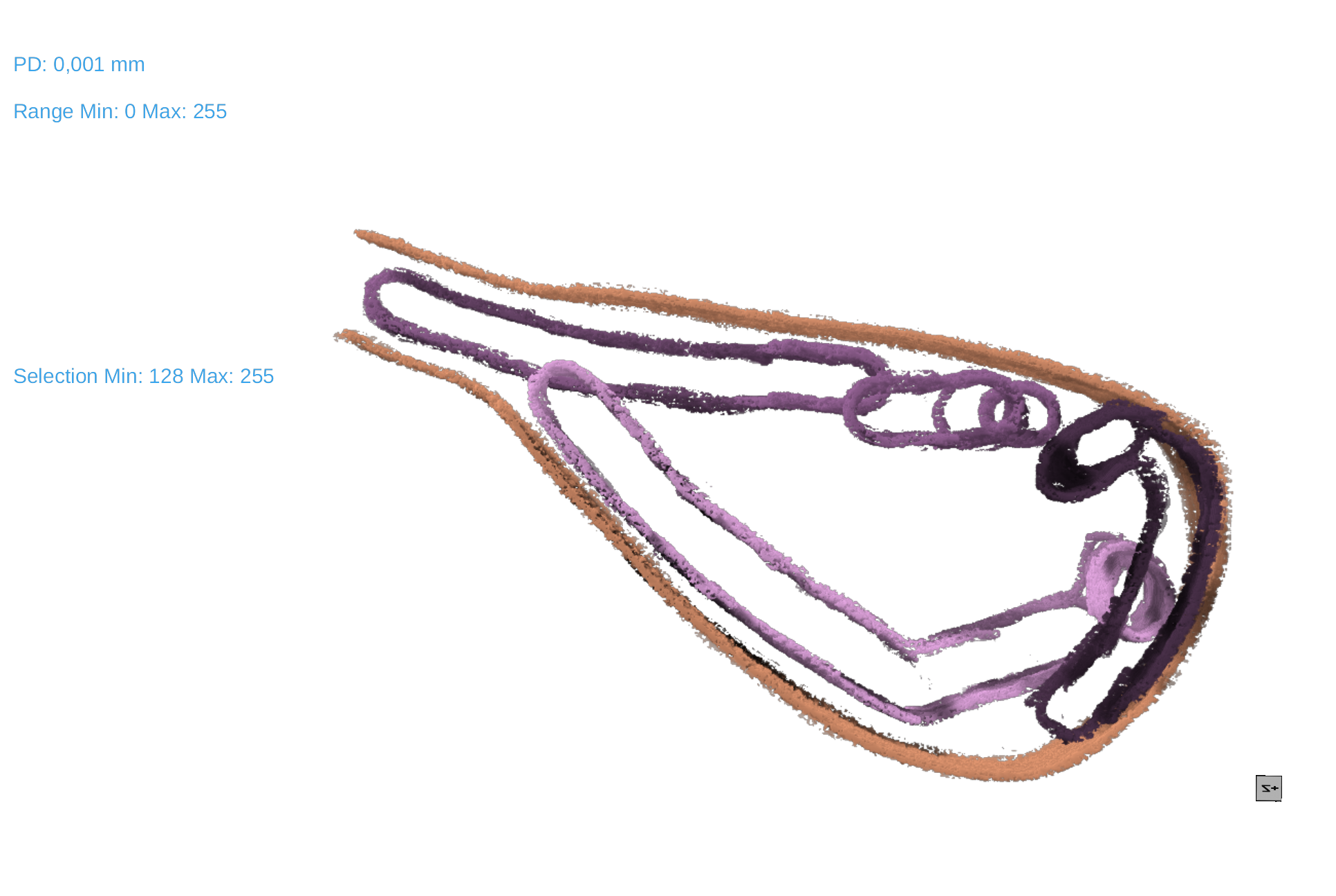

Supplement: Supplementary file 4 — Source data Fig. 3 [file 44318_2025_481_MOESM4_ESM.zip › Figure_03/3K/3K_WSN-M1-Udorn_Dragonfly.png]

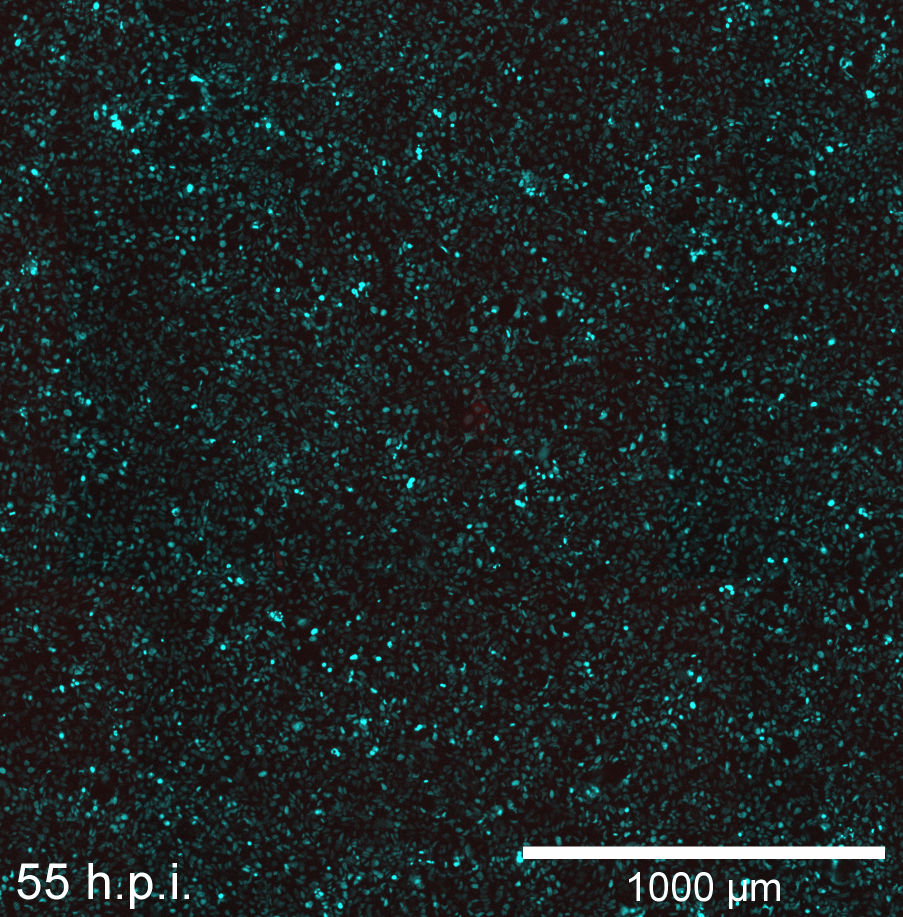

Supplement: Supplementary file 5 — Source data Fig. 4 [file 44318_2025_481_MOESM5_ESM.zip › Figure_04/4G/WSN-Calu-3-agarose1%.tif]

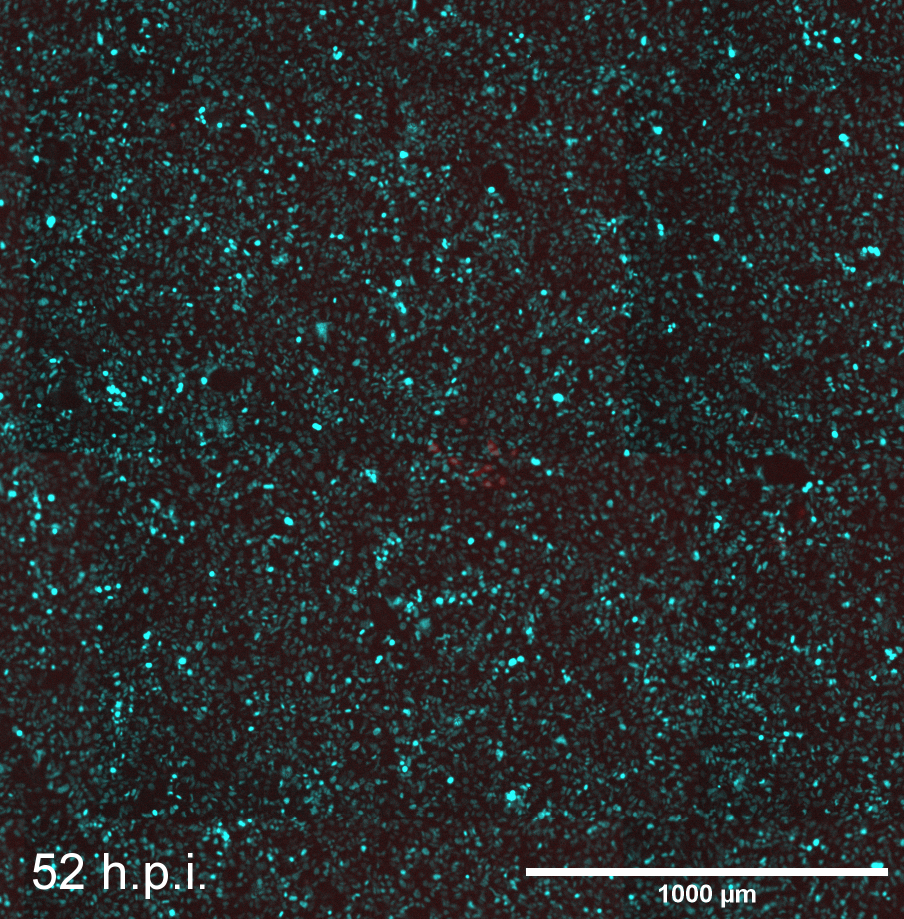

Supplement: Supplementary file 5 — Source data Fig. 4 [file 44318_2025_481_MOESM5_ESM.zip › Figure_04/4G/WSN-M1-Udorn-Calu-3-agarose1%.tif]

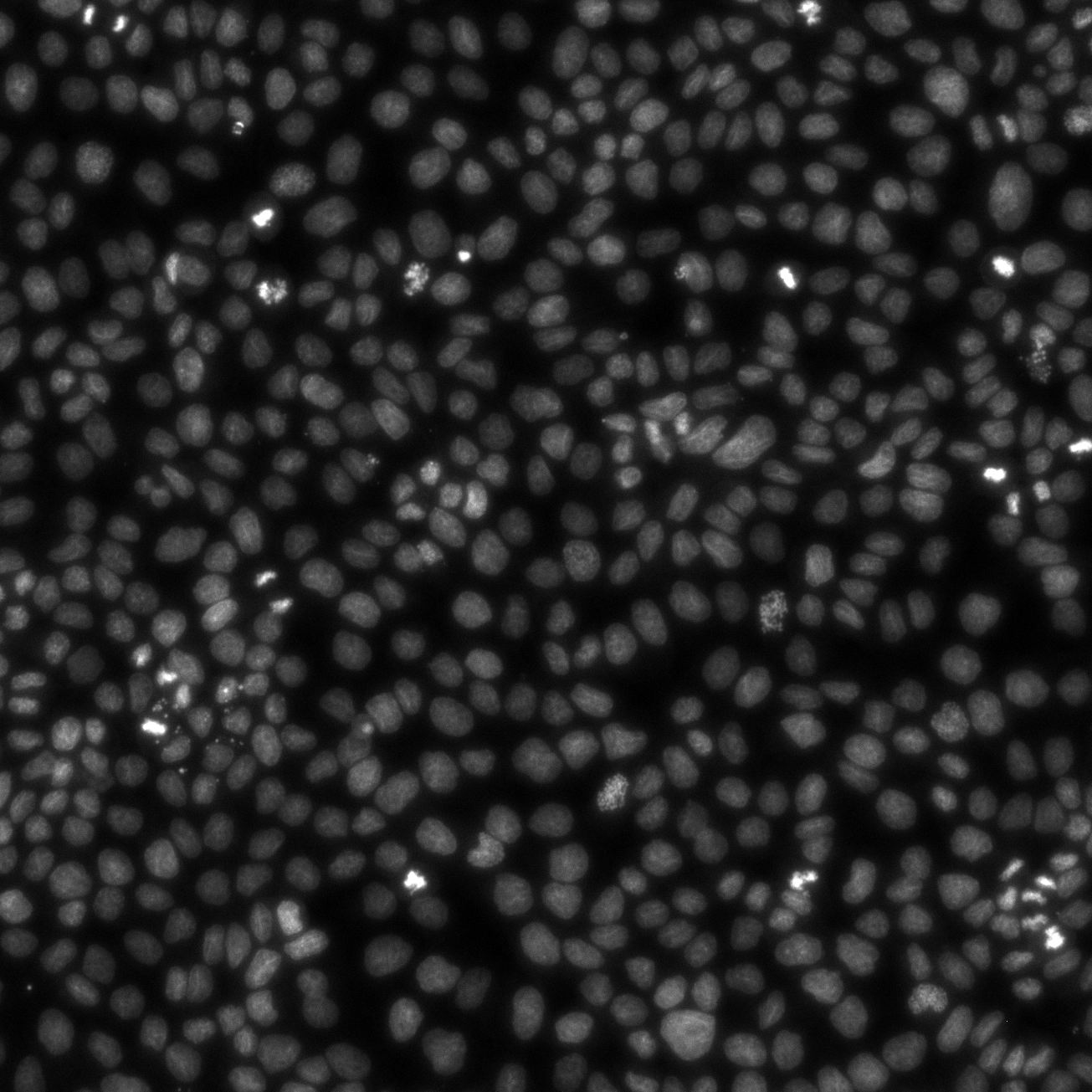

Supplement: Supplementary file 5 — Source data Fig. 4 [file 44318_2025_481_MOESM5_ESM.zip › Figure_04/4A/4A_wt_24h.tif]

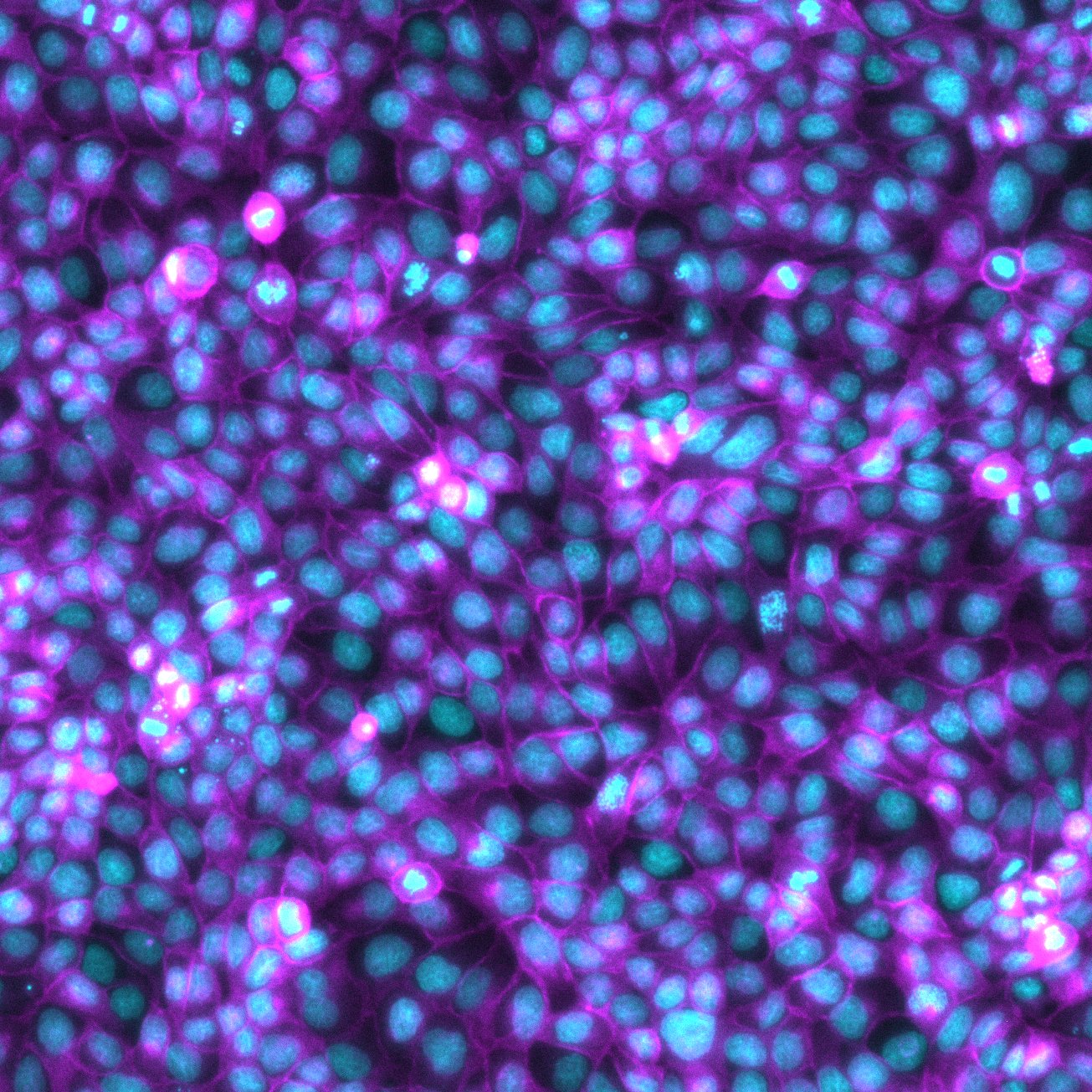

Supplement: Supplementary file 5 — Source data Fig. 4 [file 44318_2025_481_MOESM5_ESM.zip › Figure_04/4A/4A_wt_24h.jpg]

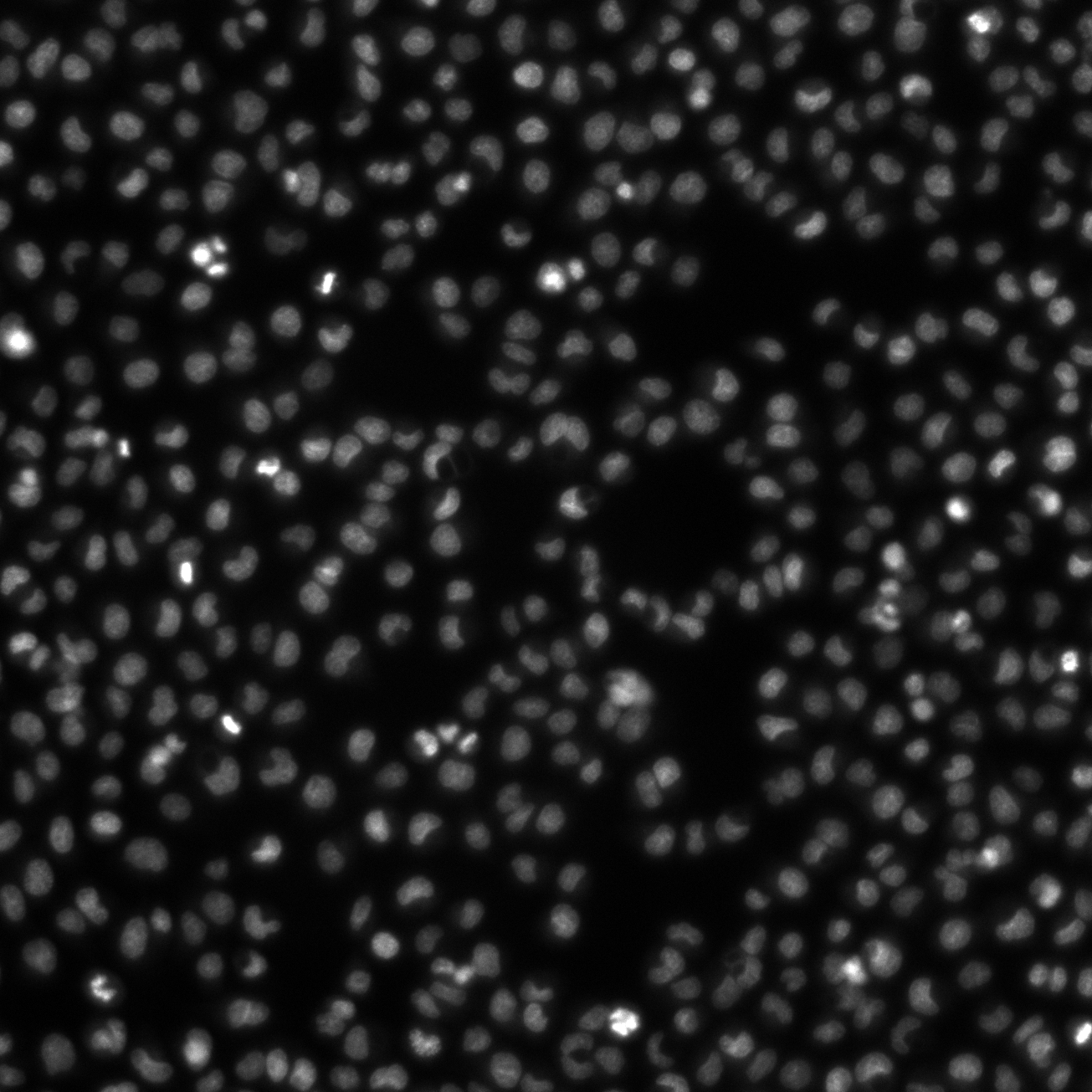

Supplement: Supplementary file 5 — Source data Fig. 4 [file 44318_2025_481_MOESM5_ESM.zip › Figure_04/4A/4A_ko_24h.tif]

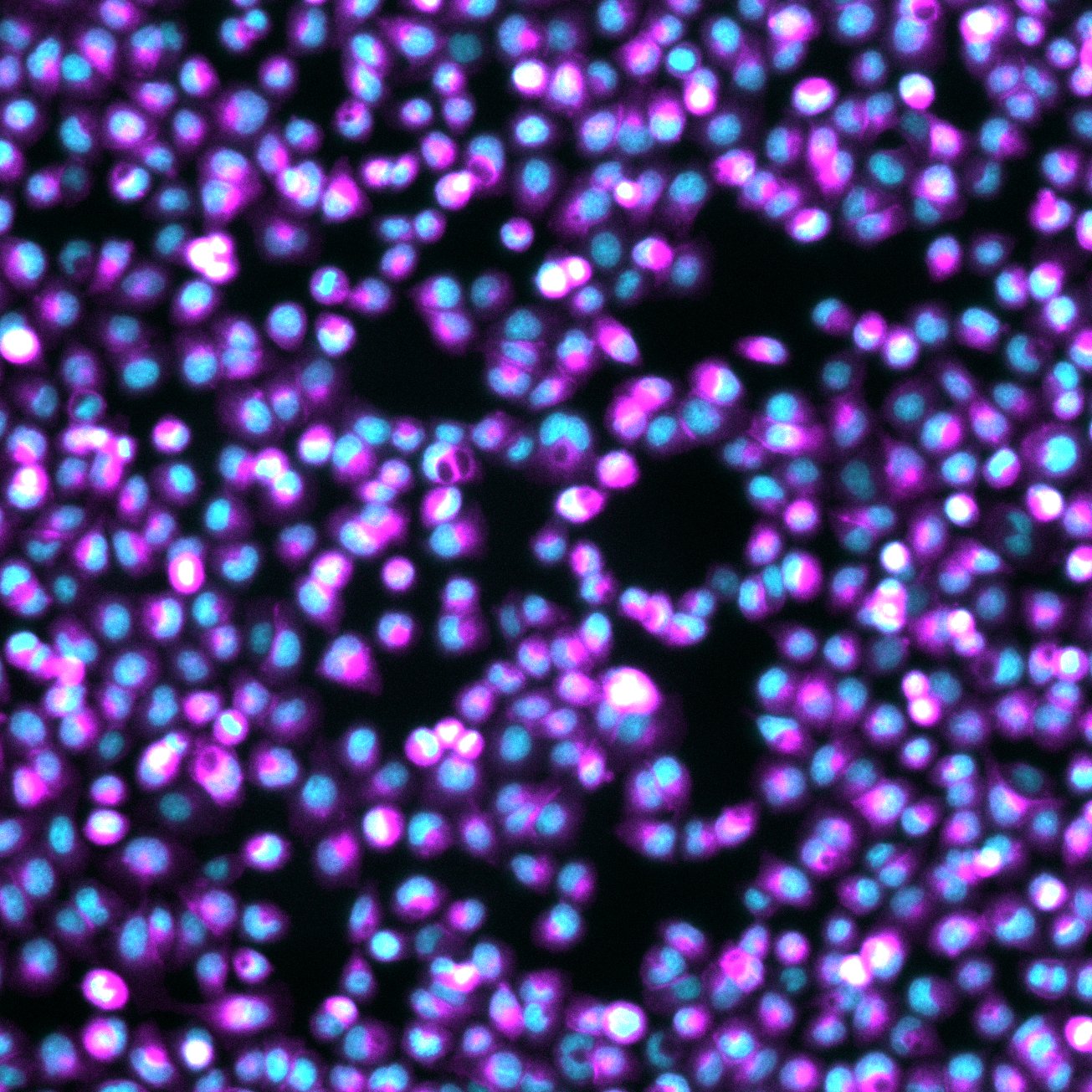

Supplement: Supplementary file 5 — Source data Fig. 4 [file 44318_2025_481_MOESM5_ESM.zip › Figure_04/4A/4A_ko_24h.jpg]

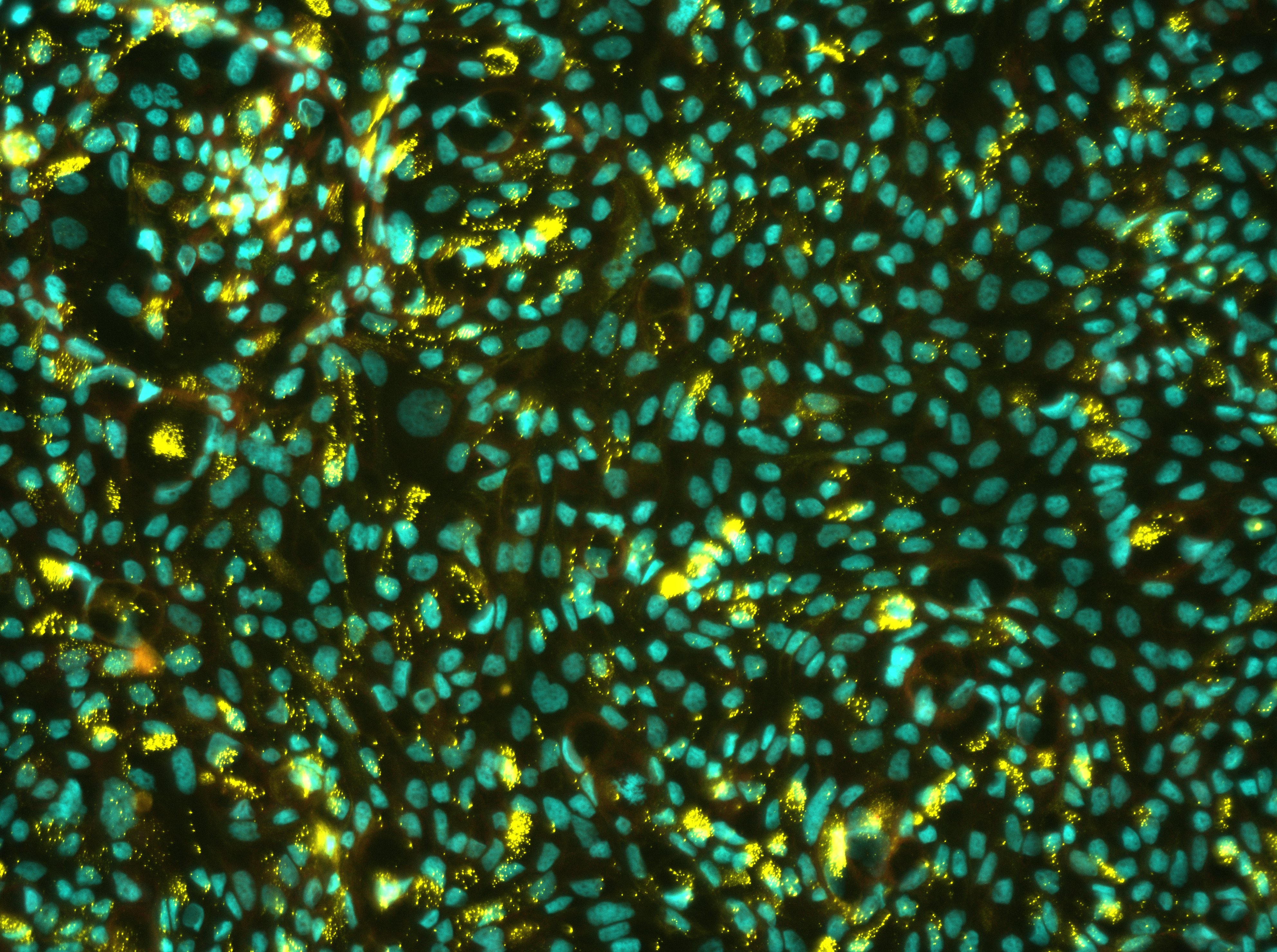

Supplement: Supplementary file 5 — Source data Fig. 4 [file 44318_2025_481_MOESM5_ESM.zip › Figure_04/4D/4D_Calu3-uninfected_muc5ac.jpg]

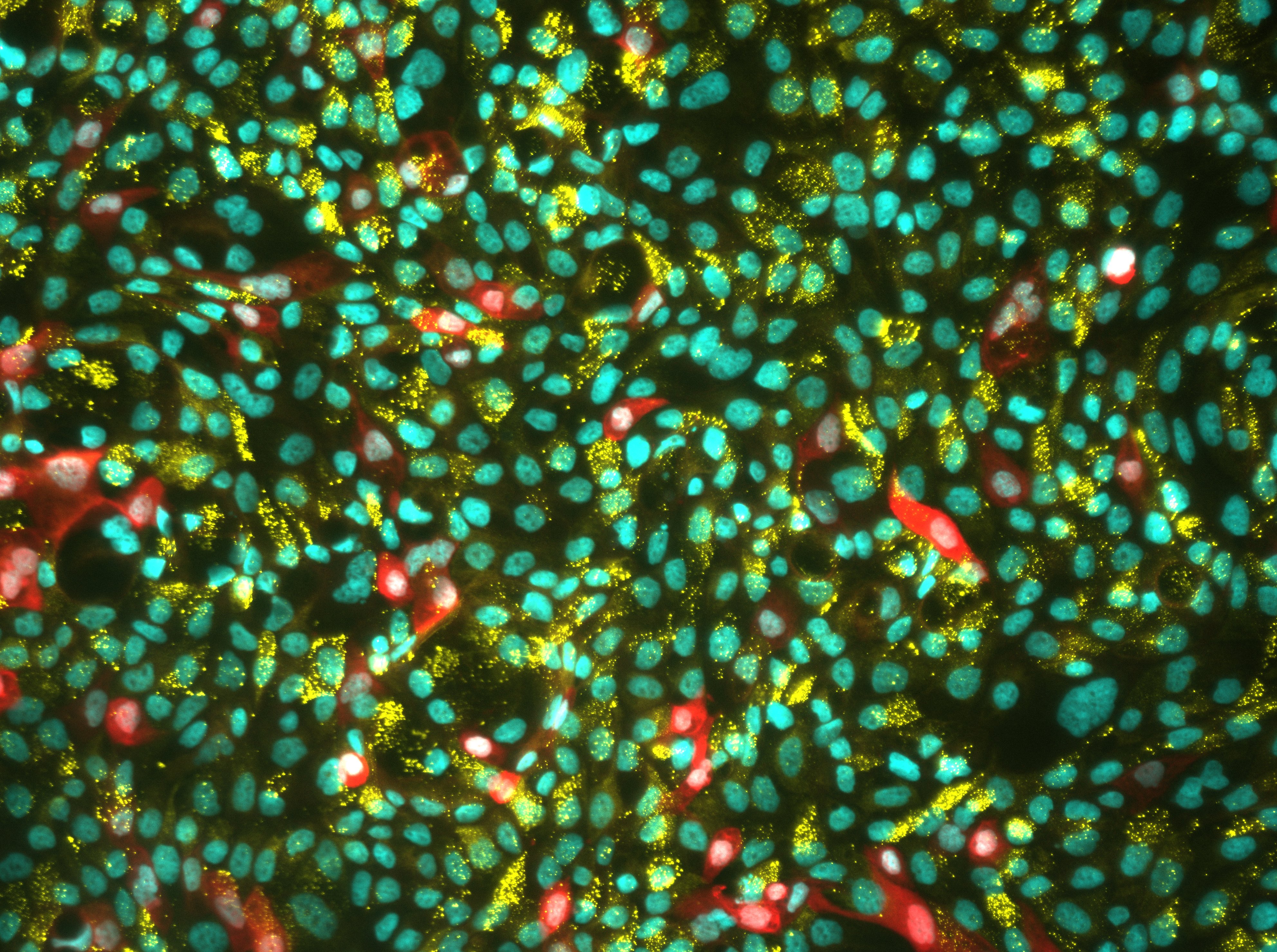

Supplement: Supplementary file 5 — Source data Fig. 4 [file 44318_2025_481_MOESM5_ESM.zip › Figure_04/4D/4D_Calu3_WSN-M1-Udorn_muc5ac.jpg]

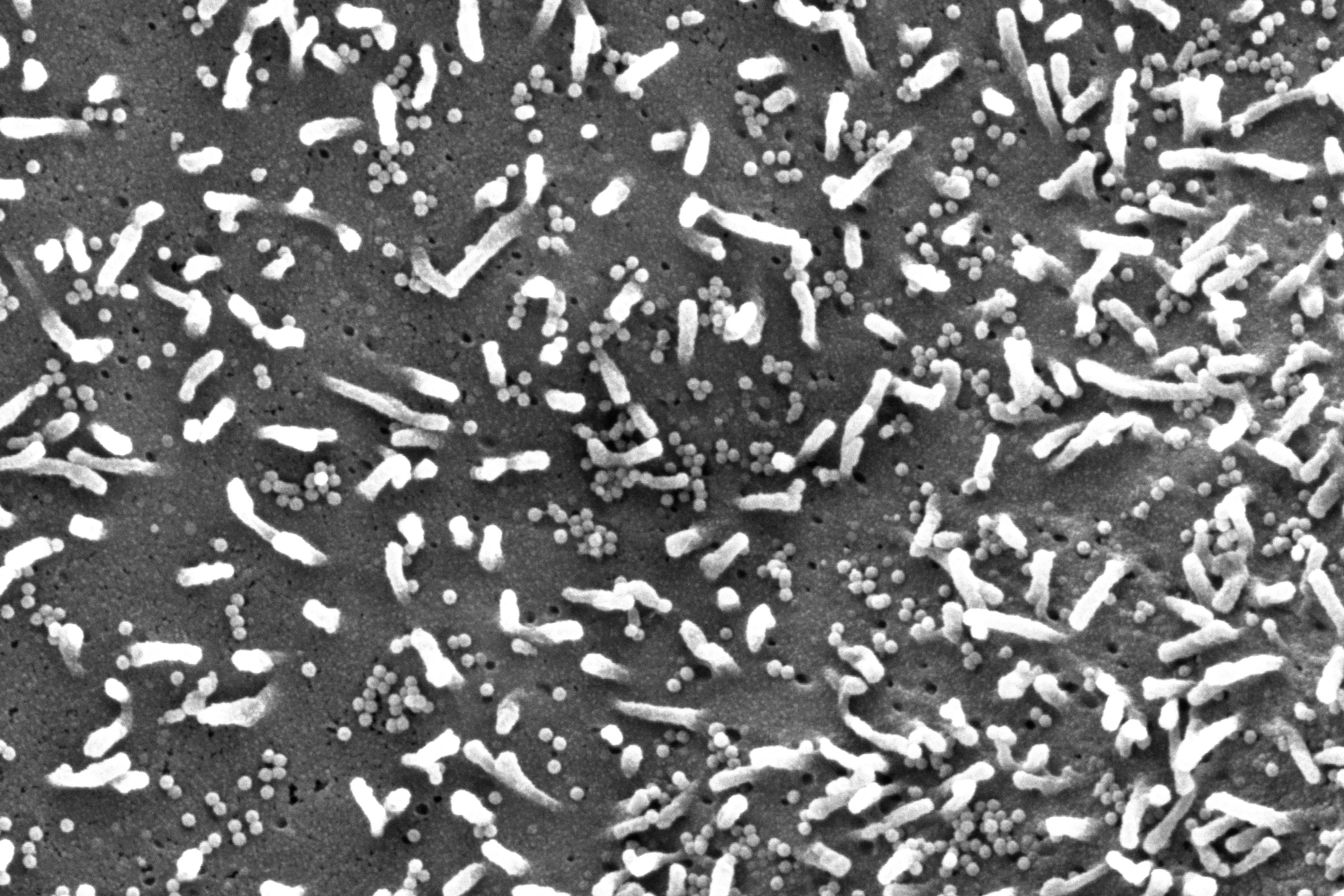

Supplement: Supplementary file 6 — Source data Fig. 5 [file 44318_2025_481_MOESM6_ESM.zip › Figure_05/5F/5F_WSN_02.tiff]

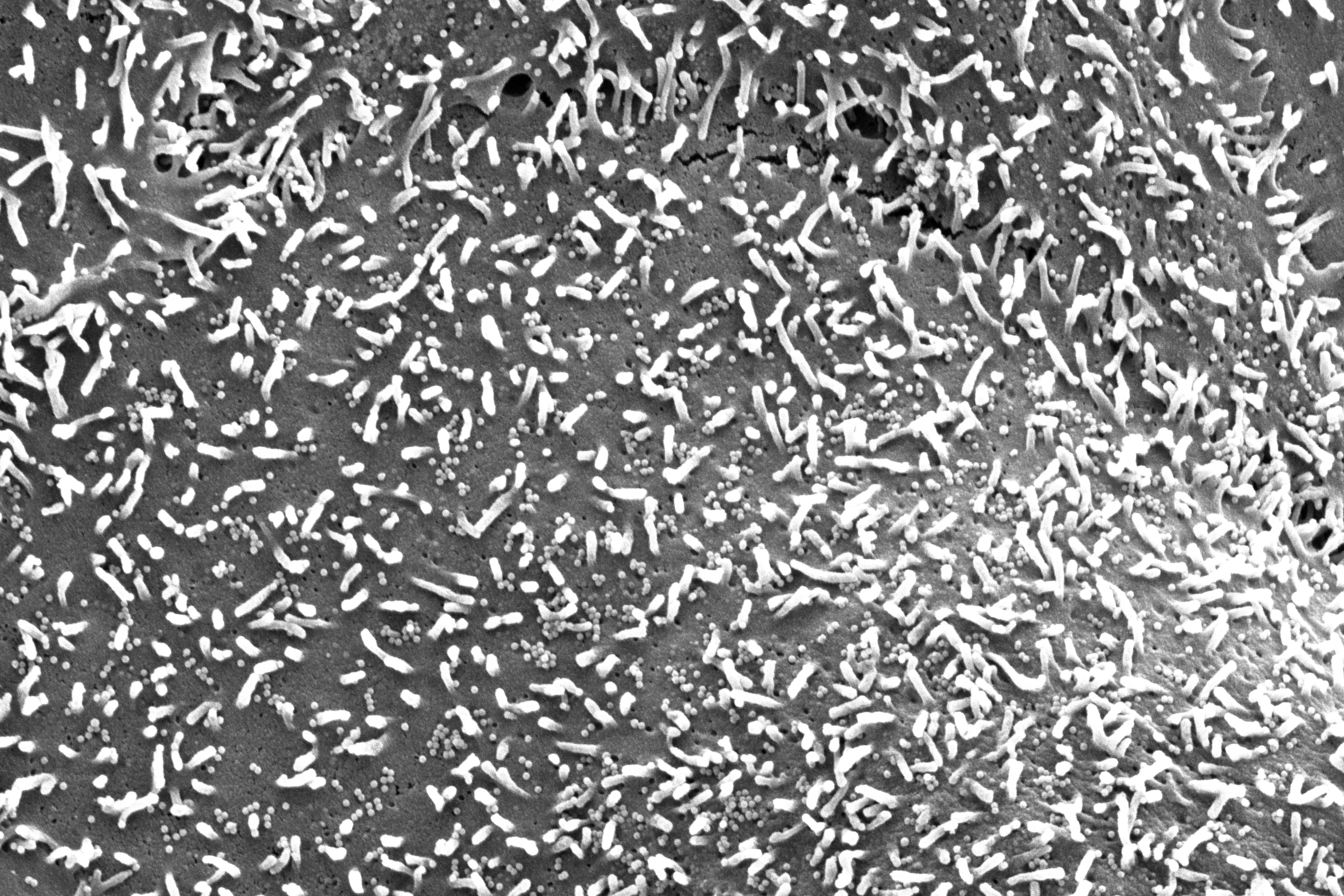

Supplement: Supplementary file 6 — Source data Fig. 5 [file 44318_2025_481_MOESM6_ESM.zip › Figure_05/5F/5F_WSN_01.tiff]

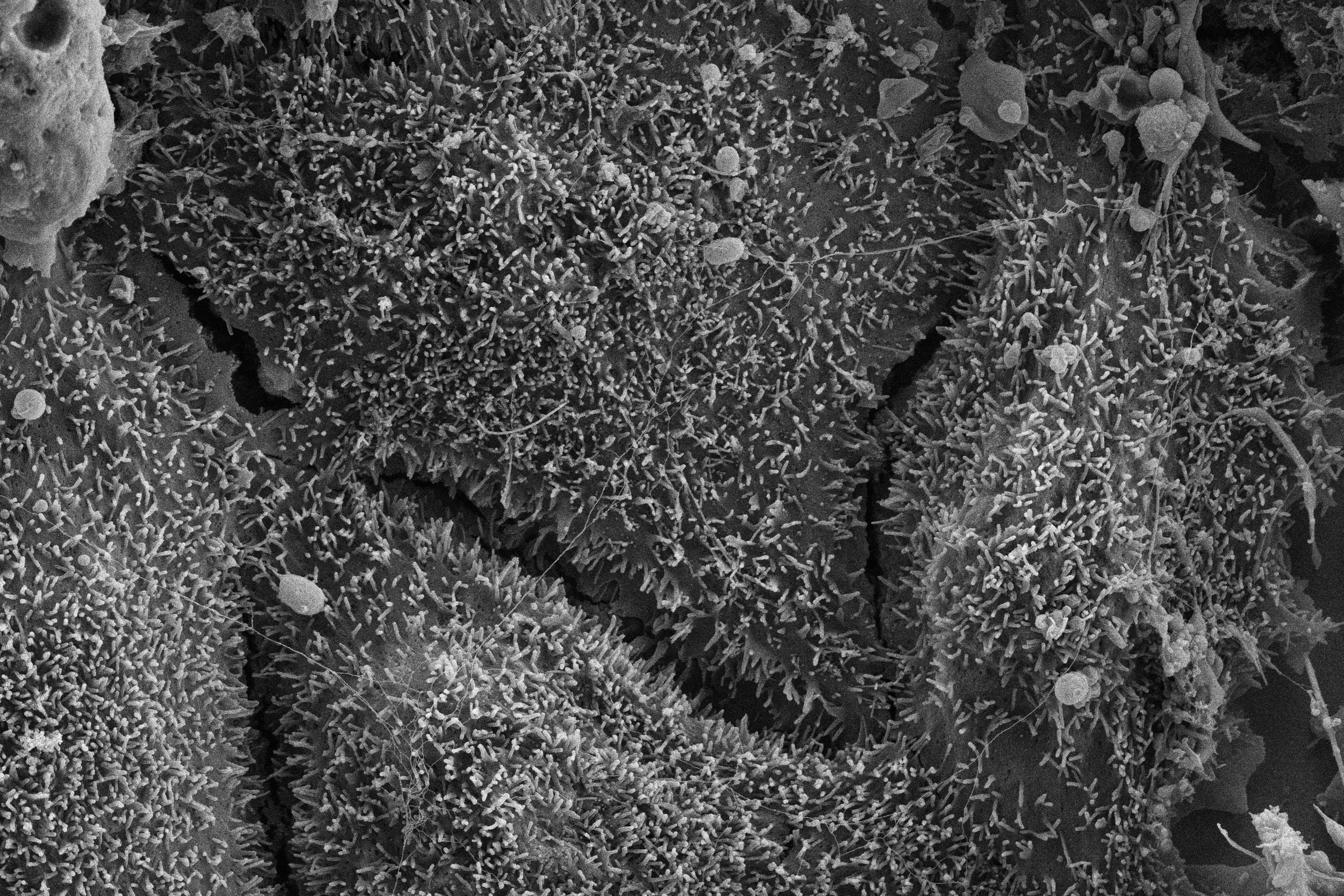

Supplement: Supplementary file 6 — Source data Fig. 5 [file 44318_2025_481_MOESM6_ESM.zip › Figure_05/5I/5I_WSN-M1-Udorn+MEDI_01.tiff]

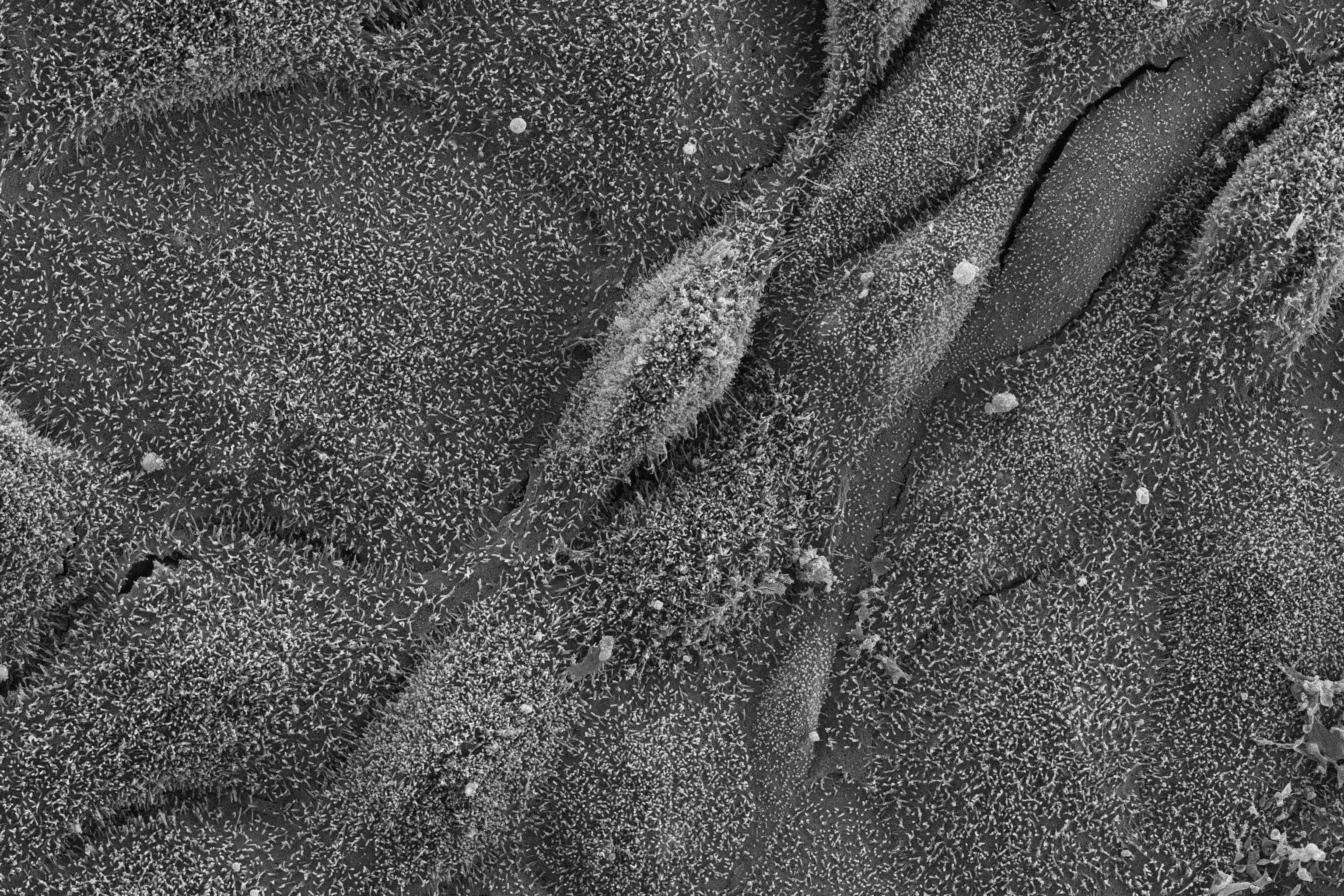

Supplement: Supplementary file 6 — Source data Fig. 5 [file 44318_2025_481_MOESM6_ESM.zip › Figure_05/5I/5I_WSN-M1-Udorn+MEDI_02.tiff]

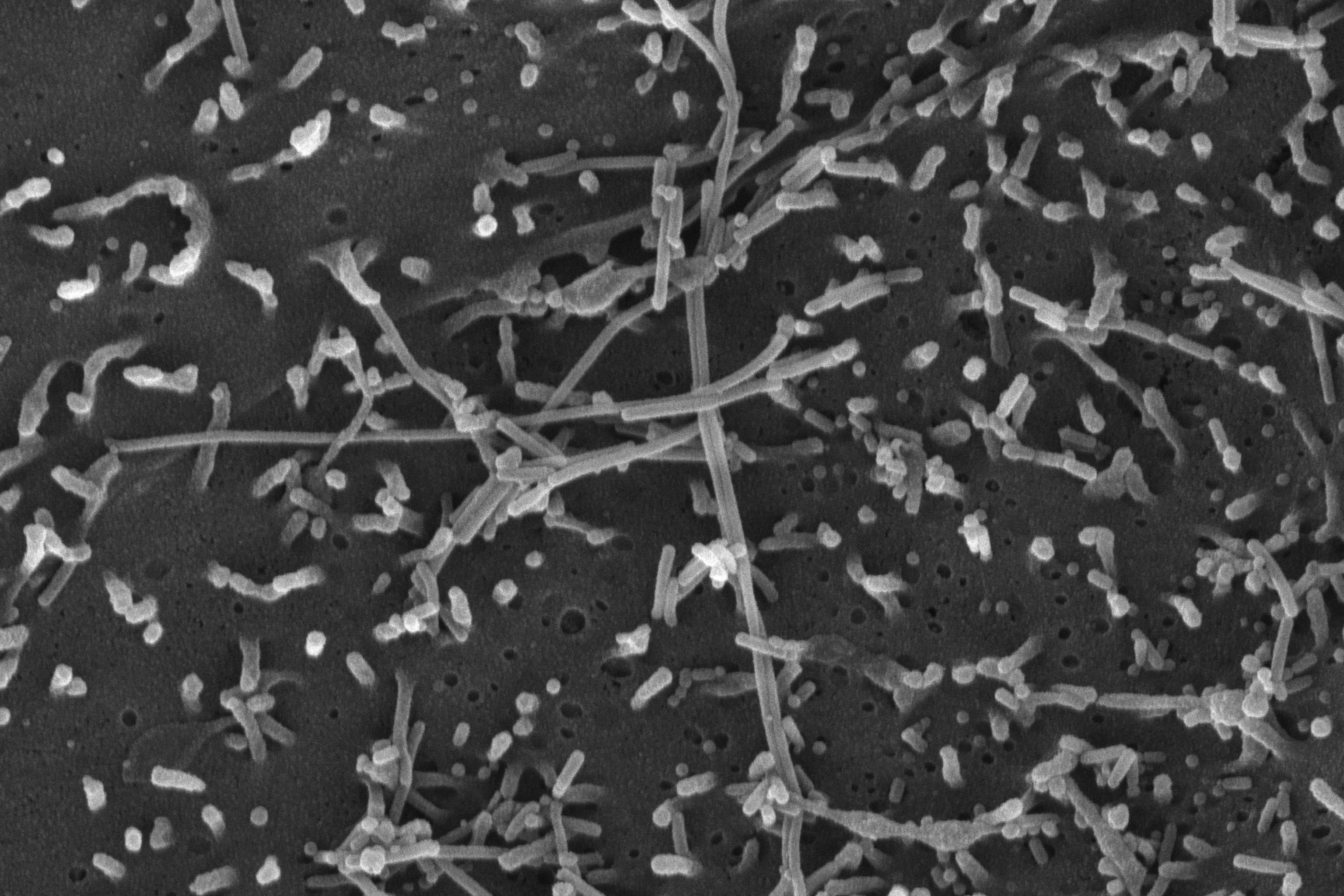

Supplement: Supplementary file 6 — Source data Fig. 5 [file 44318_2025_481_MOESM6_ESM.zip › Figure_05/5H/5H_WSN-M1-Udorn_03.tiff]

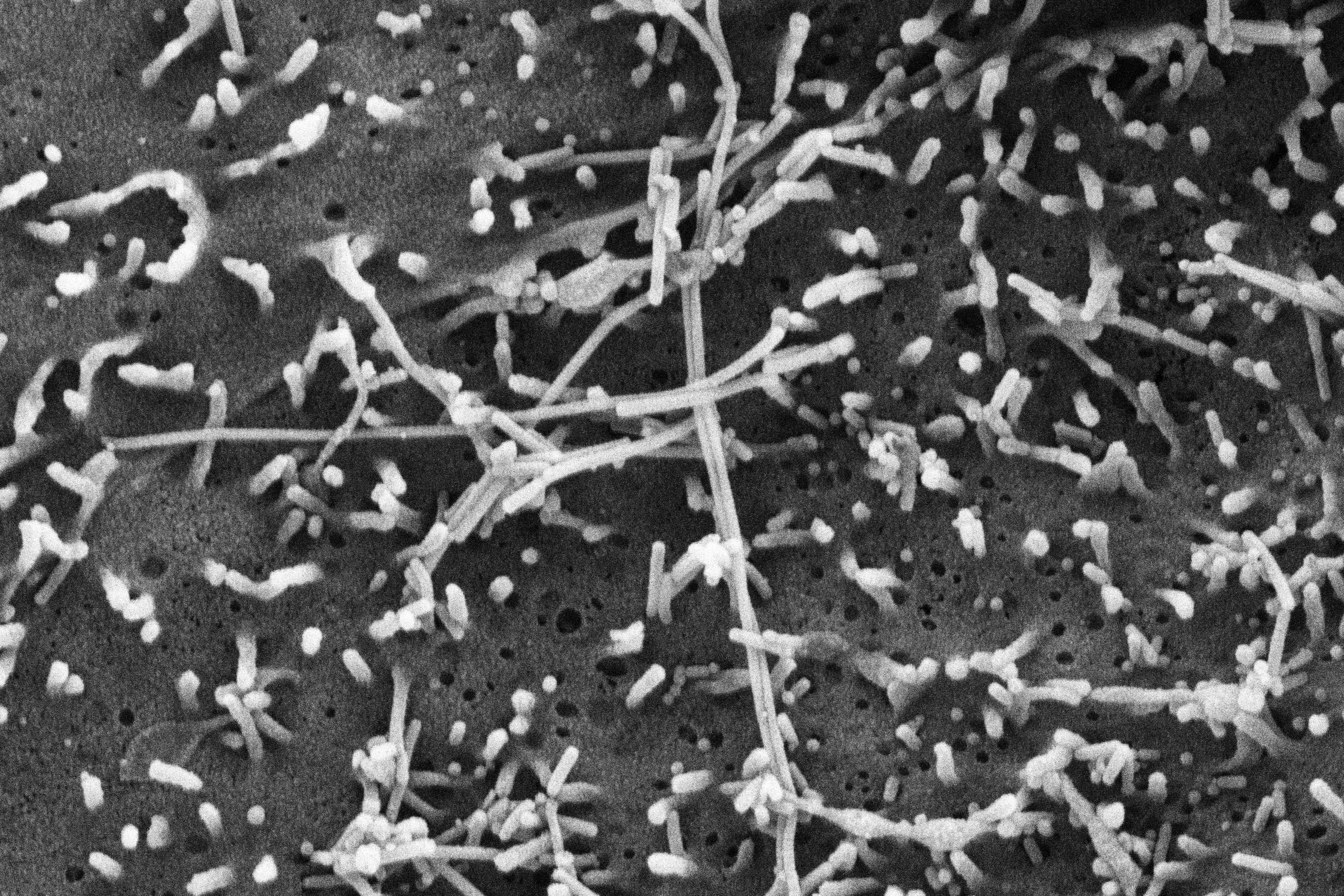

Supplement: Supplementary file 6 — Source data Fig. 5 [file 44318_2025_481_MOESM6_ESM.zip › Figure_05/5H/5H_WSN-M1-Udorn_02.tiff]

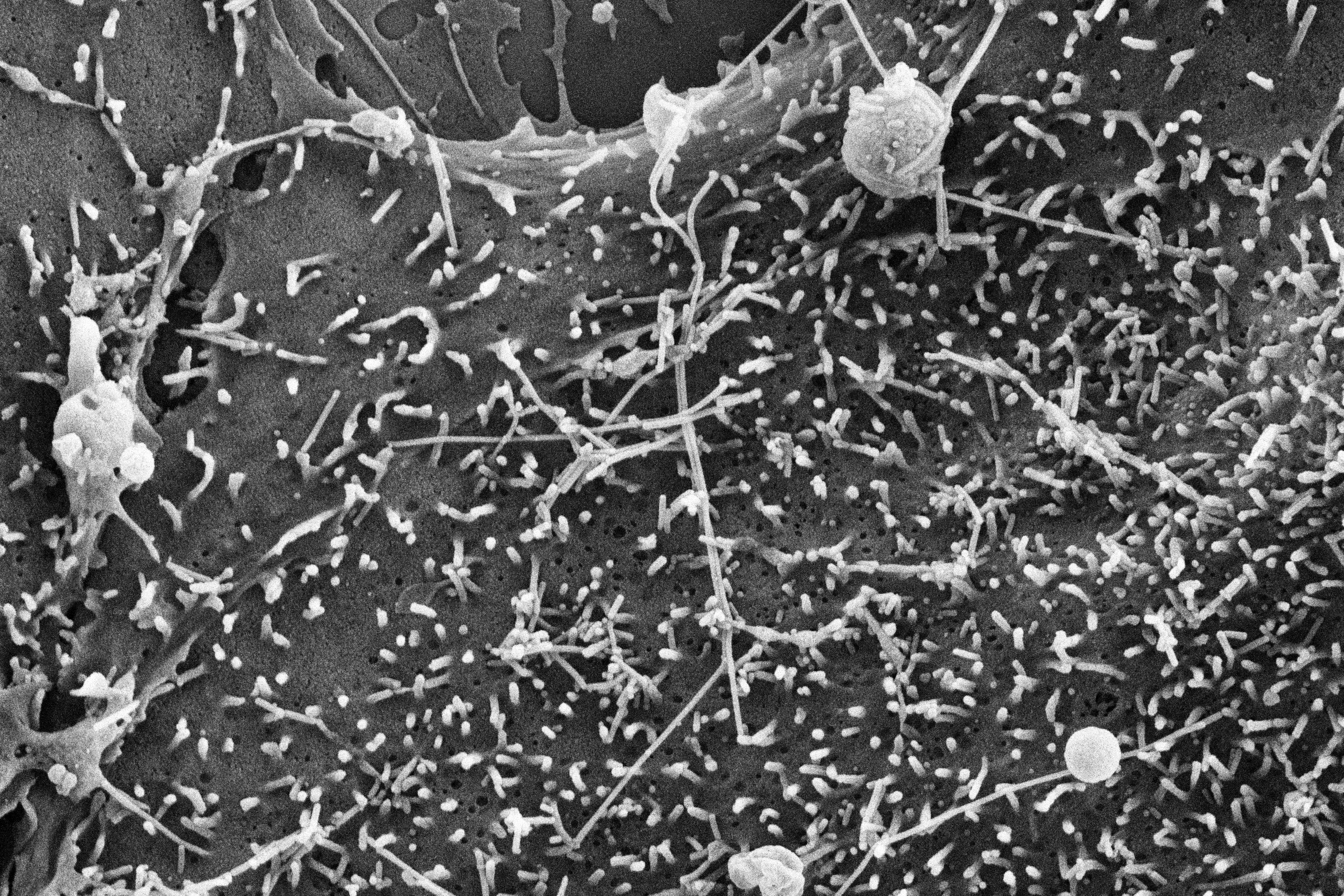

Supplement: Supplementary file 6 — Source data Fig. 5 [file 44318_2025_481_MOESM6_ESM.zip › Figure_05/5H/5H_WSN-M1-Udorn_01.tiff]

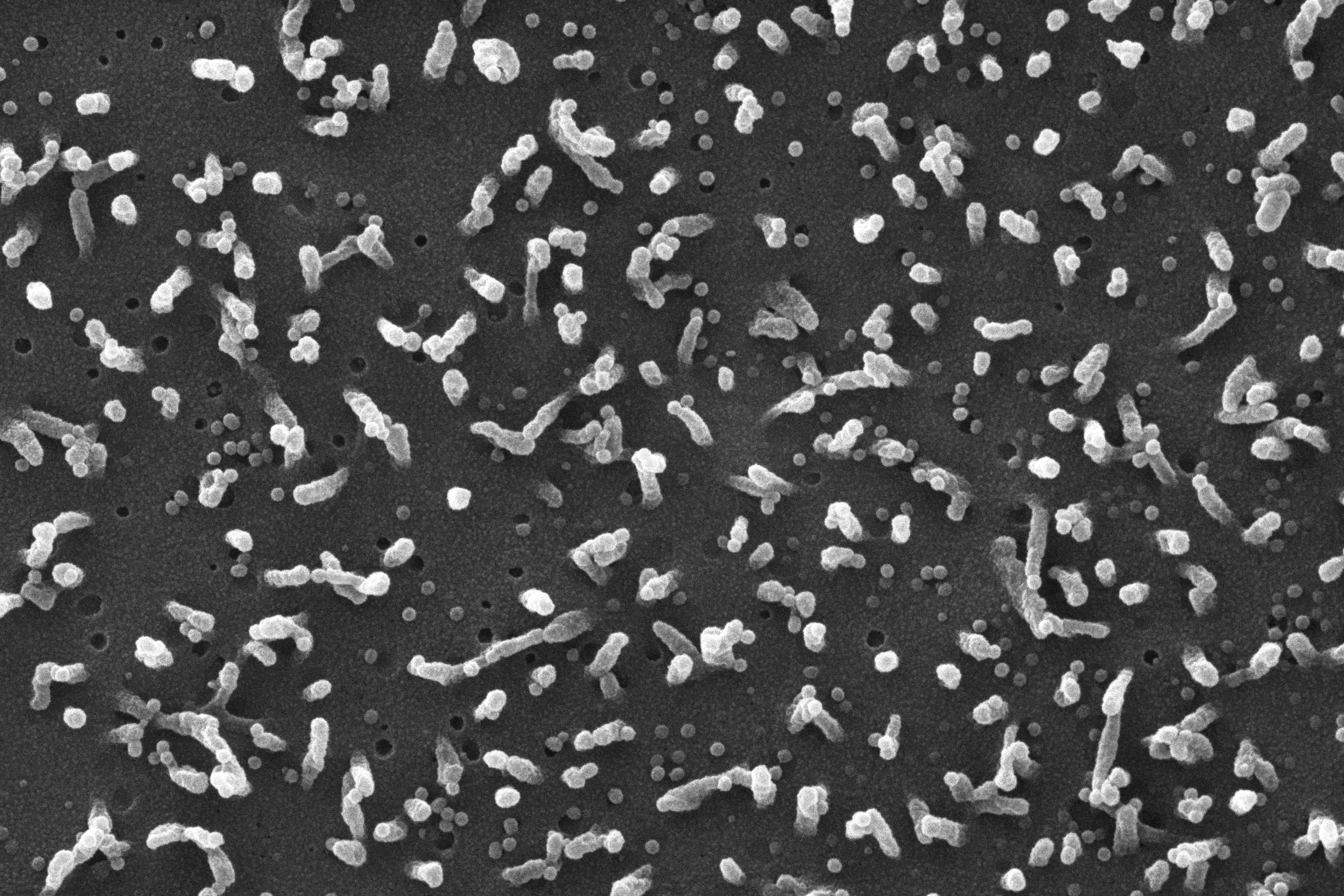

Supplement: Supplementary file 6 — Source data Fig. 5 [file 44318_2025_481_MOESM6_ESM.zip › Figure_05/5G/5G_WSN+MEDI_02.tiff]

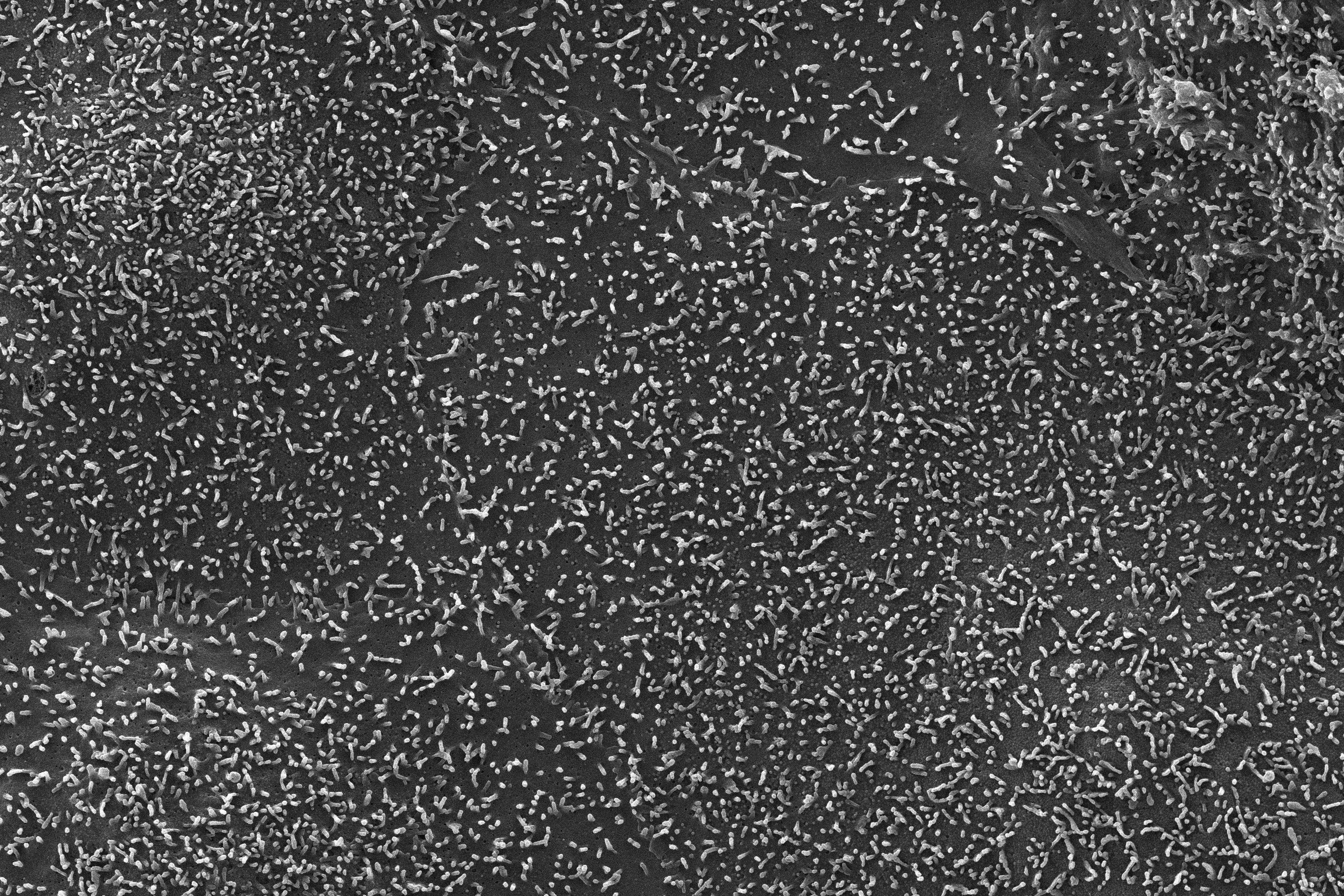

Supplement: Supplementary file 6 — Source data Fig. 5 [file 44318_2025_481_MOESM6_ESM.zip › Figure_05/5G/5G_WSN+MEDI_01.tiff]
