# Supplementary material for: Morphology-dependent entry kinetics and spread of influenza A virus
Source: EMBO J. 2025 Jun 9;44(14):3959–82. doi: 10.1038/s44318-025-00481-6 (PMC12264294; doi:10.1038/s44318-025-00481-6)
Supplement: Supplementary file 4 — Source data Fig. 3 [file 44318_2025_481_MOESM4_ESM.zip › Figure_03/3E/README_3E.docx]

Cryo-electron tomogram of A549 cell infected with WSN was deposited on EMDB.

Deposition ID: D_1292147220

Accession code: EMD-53448
