# Supplementary material for: Morphology-dependent entry kinetics and spread of influenza A virus
Source: EMBO J. 2025 Jun 9;44(14):3959–82. doi: 10.1038/s44318-025-00481-6 (PMC12264294; doi:10.1038/s44318-025-00481-6)
Supplement: Supplementary file 7 — Expanded View Figures [file 44318_2025_481_MOESM7_ESM.pdf]

Expanded View Figures

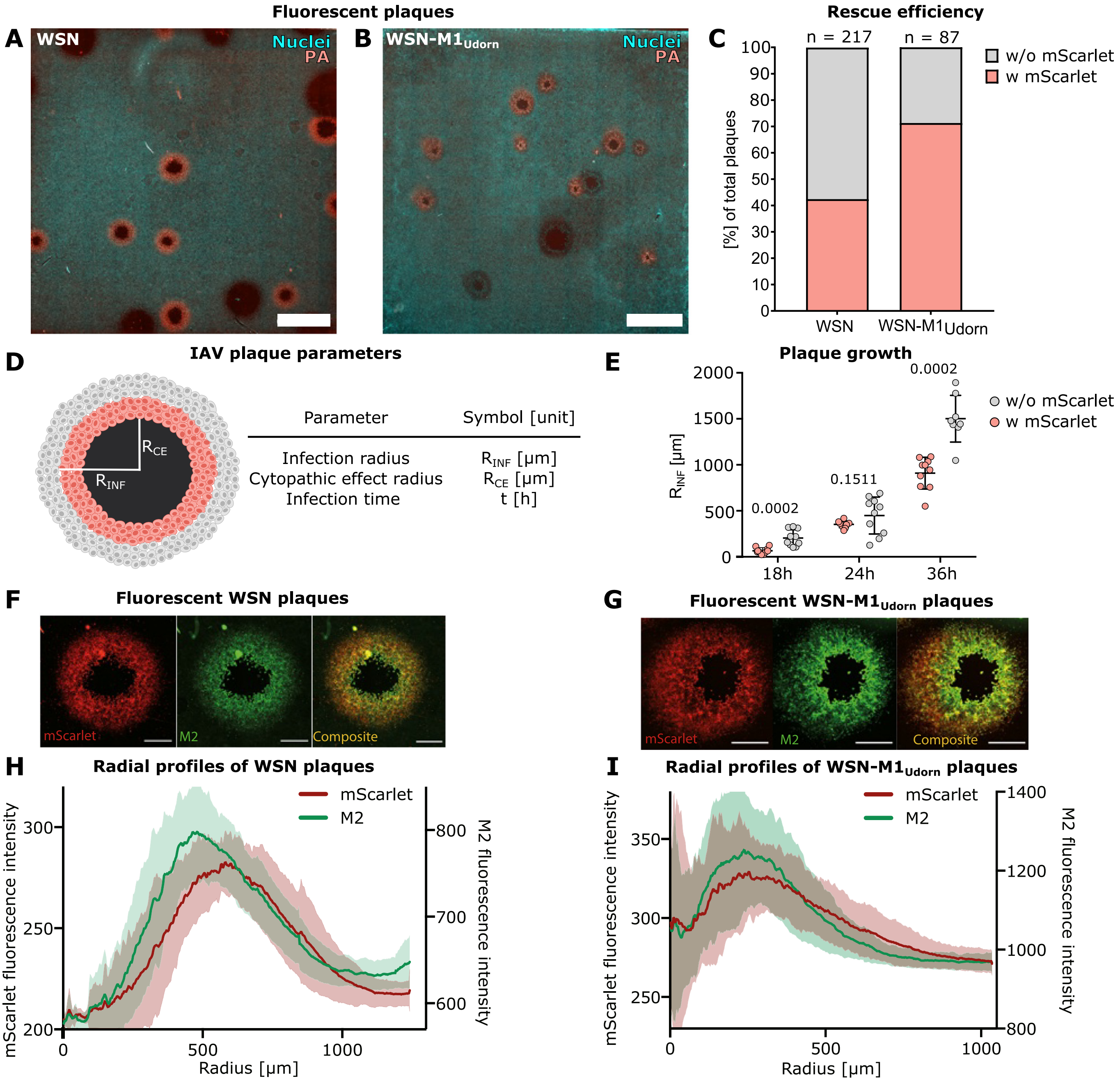

Figure EV1. Characterization of fluorescent IAV plaques.

(**A**) Exemplary images of plaques from MDCK cells infected with WSN:PAmScarlet at 36 hpi with PAmScarlet (red) and nucleus staining (cyan), scale bars: 3 mm. (**B**) Plaques from MDCK cells infected with WSN-$M1_{Udorn}$:PAmScarlet at 36 hpi with PAmScarlet (red) and nucleus staining (cyan), scale bars: 3 mm. (**C**) Percentages of plaques with (w) (red) and without (w/o) (gray) mScarlet signal for WSN:PAmScarlet ($n = 217$) and WSN-M1Udorn:PAmScarlet ($n = 87$), quantified at different time points. (**D**) Schematic representation of the zones within a fluorescent plaque and parameters for quantification of IAV spread. (**E**) Infection radius (R_{INF}) of plaques with (red) and without (gray) mScarlet quantified for 10 plaques per condition, at 18, 24, 36 hpi. Means and standard deviations are indicated. Exact *p* values for unpaired *t* tests: 2.151e-4, 1.511e-1, 2.324e-5. (**F**) Zoom-in of a fluorescent WSN:PAmScarlet plaque in MDCK cells from (**A**) with PAmScarlet (red), immunostained M2 (green), and a composite of both signals (yellow). The panel was intentionally duplicated from Fig. 1G to facilitate comparison between PAmScarlet and M2 stainings, scale bar: 500 µm. (**G**) Exemplary images of a fluorescent WSN-$M1_{Udorn}$:PAmScarlet plaque in MDCK cells with PAmScarlet (red), immunostained M2 (green), and a composite of both signals (yellow), scale bar: 500 µm. (**H**) Mean fluorescence intensities of PAmScarlet (red) and M2 (green) from radial profiles of 10 WSN:PAmScarlet plaques in MDCK cells. Standard deviations are indicated. (**I**) Mean fluorescence intensities of PAmScarlet (red) and M2 (green) from radial profiles of 10 WSN-$M1_{Udorn}$:PAmScarlet plaques in MDCK cells. Standard deviations are indicated.

© The Author(s)

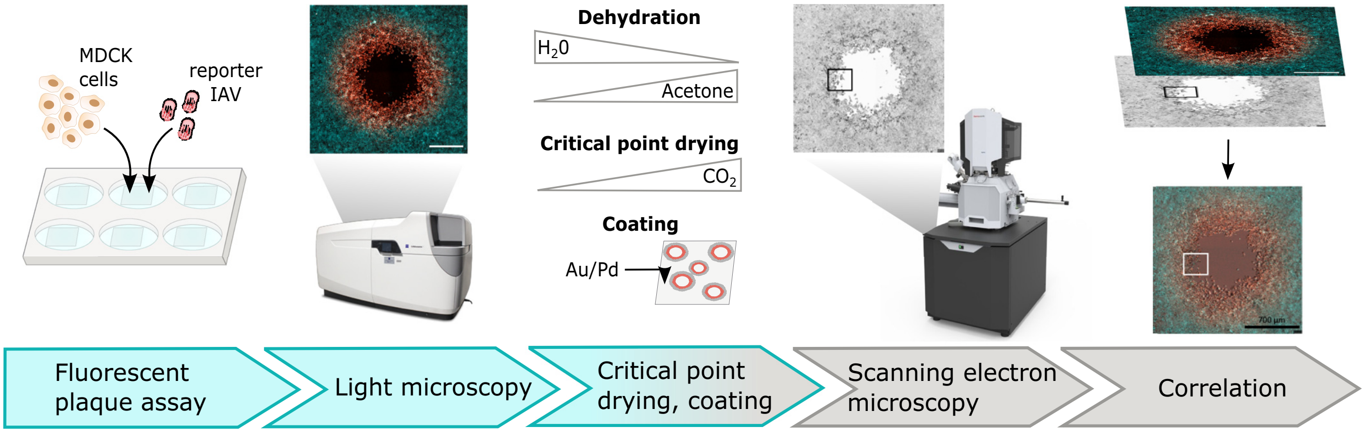

Figure EV2. Workflow of correlative light and scanning electron microscopy (CLSEM) for the study of plaque growth and IAV morphology.

MDCK cells were seeded onto ITO-coated coverslips and infected with spherical or filamentous reporter IAV expressing PAmScarlet. Plaques were imaged by fluorescence microscopy. The plaque from Fig. 2A was reused here to demonstrate the CLSEM workflow. Samples were prepared for scanning electron microscopy (SEM) by dehydration with increasing acetone concentrations. Critical point drying and sputter coating with Au/Pd was performed. SEM overview maps of plaques were acquired for correlation with fluorescent images. High-magnification SEM images were acquired.

 © The Author(s)

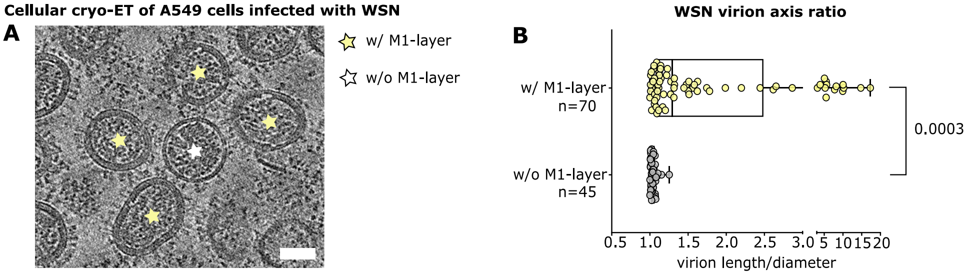

Figure EV3. Cellular cryo-ET of IAV-infected A549 cells.

(**A**) Slice through a cryo-electron tomogram showing spherical WSN particles in endosomal compartments of an infected A549 cell at 15–30 min post infection.Virions with assembled matrix protein 1 layer (w/ M1-layer) are highlighted with yellow stars. One virion without (w/o) M1-layer is highlighted with a white star, scale bar: 50 nm. (**B**) WSN virion length/diameter ratio of particles w/ M1-layer (yellow) and w/o M1-layer (gray) within endosomes of infected A549 cells, quantified from cryo-electron tomograms. Boxes show quartiles with median lines and min to max values. Significance analysis was done by Mann–Whitney *U*-test. Exact *p* value: 3.2e-4.

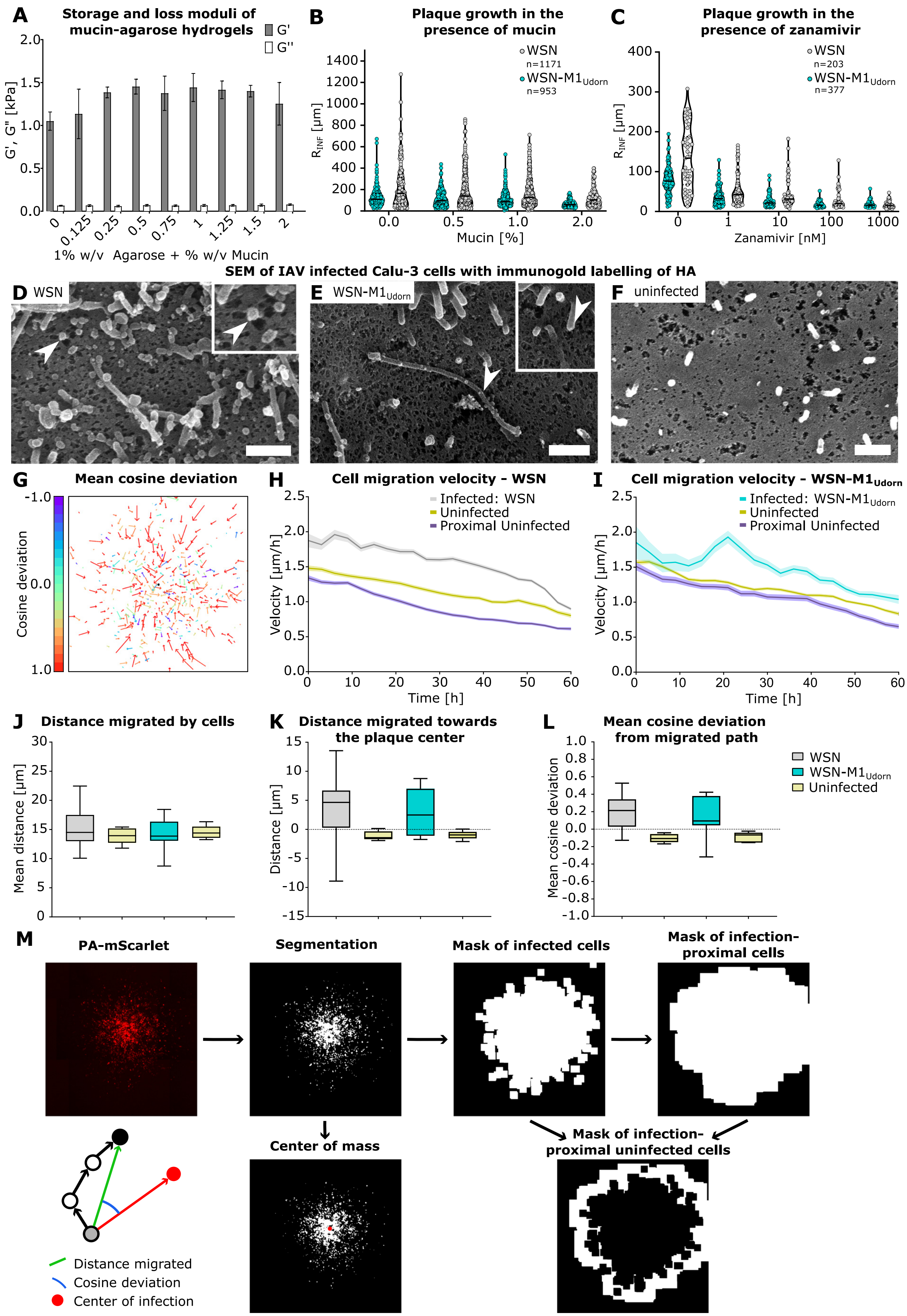
A
Storage and loss moduli of mucin-agarose hydrogels
G'
G''
G', G'' [kPa]
2.0
1.5
1.0
0.5
0.0
0
0.125
0.25
0.5
0.75
1
1.25
1.5
2
1% w/v Agarose + % w/v Mucin
B
Plaque growth in the presence of mucin
WSN n=1171
WSN-M1Udorn n=953
RINF [µm]
1400
1200
1000
800
600
400
200
0
0.0
0.5
1.0
2.0
Mucin [%]
C
Plaque growth in the presence of zanamivir
WSN n=203
WSN-M1Udorn n=377
RINF [µm]
400
300
200
100
0
0
1
10
100
1000
Zanamivir [nM]
SEM of IAV infected Calu-3 cells with immunogold labelling of HA
D WSN
E WSN-M1Udorn
F uninfected
G
Mean cosine deviation
Cosine deviation
-1.0
0.0
1.0
H
Cell migration velocity - WSN
Infected: WSN
Uninfected
Proximal Uninfected
Velocity [µm/h]
2.5
2.0
1.5
1.0
0.5
0.0
0
10
20
30
40
50
60
Time [h]
I
Cell migration velocity - WSN-M1Udorn
Infected: WSN-M1Udorn
Uninfected
Proximal Uninfected
Velocity [µm/h]
Time [h]
J
Distance migrated by cells
Mean distance [µm]
30
25
20
15
10
5
0
K
Distance migrated towards the plaque center
Distance [µm]
15
10
5
0
-5
-10
-15
L
Mean cosine deviation from migrated path
Mean cosine deviation
1.0
0.8
0.6
0.4
0.2
0.0
-0.2
-0.4
-0.6
-0.8
-1.0
WSN
WSN-M1Udorn
Uninfected
M
PA-mScarlet
Segmentation
Mask of infected cells
Mask of infection-proximal cells
Center of mass
Mask of infection-proximal uninfected cells
Distance migrated
Cosine deviation
Center of infection

 © The Author(s)

Figure EV4. IAV plaque growth in the presence of mucin or zanamivir, immunoelectron microscopy of IAV and Calu-3 cell migration.

(**A**) Rheological properties of agarose hydrogels in the presence of increasing mucin concentrations. The storage modulus (G', gray) reflects elasticity and the loss modulus (G'', white) represents viscosity. Bars show averages of 3–4 repeated measurements in 5 different mucin-agarose hydrogels with indicated standard deviations. (**B**) R_{INF} of WSN (gray) and WSN-$M1_{Udorn}$ (cyan) in the presence of increasing mucin concentrations at 36 hpi in MDCK cells. (**C**) R_{INF} of WSN (gray) and WSN-$M1_{Udorn}$ (cyan) in the presence of increasing zanamivir concentrations at 36 hpi in MDCK cells. (**D**) Scanning electron microscopy (SEM) of Calu-3 cells infected with WSN:PAmScarlet, scale bar: 500 nm. (**E**) SEM of Calu-3 cells infected with WSN-$M1_{Udorn}$:PAmScarlet, fixed at 4 days post infection. White arrowheads indicate 20 nm immunogold labeling of hemagglutinin (HA), scale bar: 500 nm. (**F**) SEM of uninfected Calu-3 cells, scale bar: 500 nm. (**G**) Migrated paths of Calu-3 cells tracked from their first to last position of time-lapse movies. Color code of arrows represent the cosine deviation from migrated path, where $+1$ indicates migration to the plaque center and -1 indicates migration away from the center. (**H**) Mean velocity of cell migration trajectories for WSN:PAmScarlet infected and uninfected Calu-3 cells. Standard errors are indicated. The time point 0 h corresponds to 52 hpi. (**I**) Mean velocity of cell migration trajectories for WSN-$M1_{Udorn}$:PAmScarlet infected and uninfected Calu-3 cells. Standard errors are indicated. The time point 0 h corresponds to 52 hpi. (**J**) Mean migrated distance of Calu-3 cells from their first to last position. Boxes show quartiles with median lines and min to max values. (**K**) Migrated distance of Calu-3 cells towards the focus center. For each movie, the distances are averaged over all trajectories. Boxes show quartiles with median lines and min to max values. (**L**) Mean cosine deviation of cell movement from migrated path with positive values indicating migration towards the center of infection. Boxes show quartiles with median lines and min to max values. Color code of plots H-M: gray: infected with WSN:PAmScarlet, cyan: infected with WSN-$M1_{Udorn}$:PAmScarlet, yellow: uninfected cells, blue: proximal uninfected cells. (**M**) Workflow of tracking and motion analysis for cells within IAV foci, using foci from Fig. 4G as an example. IAV-infected cells expressing PAmScarlet (red) were segmented by adaptive thresholding. From the last segmented image of the time-lapse series, the center of infection was determined as the center of mass. Based on the segmentation, binary masks of infection foci were created by binary dilation and hole filling. A mask for infection-proximal cells was created by further dilating this mask 120 times. To obtain a mask for uninfected cells in proximity to IAV foci the mask of infected cells was subtracted from the mask of infection-proximal cells. Cell migration was determined by probabilistic particle tracking. The distance migrated from the first to the last position (green) and the cosine of the angle (blue) between the directions from the first to the last position (green) and the first position and the plaque center (red) were computed.

© The Author(s)

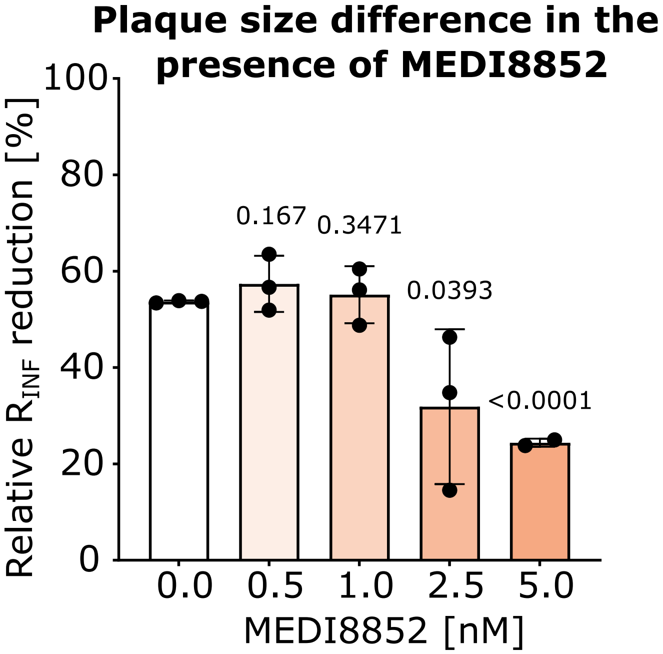

Figure EV5. Effect of neutralizing MEDI8852 antibodies on IAV plaque size.

Reduction of infection radius (R_{INF}) of WSN-M1$_{Udorn}$ plaques relative to WSN plaques in MDCK cells in the presence of indicated MEDI8852 concentrations. Relative reduction [%] = 100 − (Radius of WSN-M1$_{Udorn}$/Radius of WSN × 100). Mean and standard deviations are indicated for three independent experiments. *P* values were calculated using one-sided Student's *t* test comparing each concentration with the untreated control. Exact *p* values: 1.670e-1, 3.471e-1, 3.930e-2, 4.803e-6.

 © The Author(s)
